# DKK3 Initially Preserves Acinar Integrity Through MEK‐Fos Signaling, but Later Switches to an Oncogenic Role in Pancreatic Cancer

**DOI:** 10.1002/advs.202417606

**Published:** 2025-10-28

**Authors:** Dharini Srinivasan, Elodie Roger, Lukas Perkhofer, Eleni Zimmer, Thomas F.E. Barth, Julia P. Mosler, Anna Härle, Stephanie E. Weissinger, Nadine T. Gaisa, Peter Möller, Nico Fischer, Chantal Allgöwer, J.‐Matthias Löhr, Dirk Grimm, Thomas Seufferlein, Stefan Liebau, Ninel Azoitei, Michael K. Melzer, Frank Arnold, Johann Gout, Alexander Kleger

**Affiliations:** ^1^ Institute for Molecular Oncology and Stem Cell Biology Ulm University Hospital 89081 Ulm Germany; ^2^ Division of Interdisciplinary Pancreatology Department of Internal Medicine I Ulm University Hospital 89081 Ulm Germany; ^3^ Institute of Pathology Ulm University Hospital 89081 Ulm Germany; ^4^ Department of Infectious Diseases/Virology Section Viral Vector Technologies Medical Faculty Heidelberg University 69120 Heidelberg Germany; ^5^ BioQuant BQ0030 Heidelberg University 69120 Heidelberg Germany; ^6^ Department of Clinical Science Intervention and Technology (CLINTEC) Pancreas Cancer Research Lab Karolinska Institute 141 52 Stockholm Sweden; ^7^ German Center for Infection Research (DZIF) and German Center for Cardiovascular Research (DZHK) partner site Heidelberg 69120 Heidelberg Germany; ^8^ Department of Internal Medicine I Ulm University Hospital 89081 Ulm Germany; ^9^ Institute of Neuroanatomy & Developmental Biology INDB 72074 Tübingen Germany; ^10^ Department of Urology Ulm University Hospital 89081 Ulm Germany; ^11^ Cancer Biology & Genetics Program Memorial Sloan Kettering Cancer Center New York NY 10065 USA; ^12^ Core Facility Organoids Ulm University 89081 Ulm Germany

**Keywords:** DKK3, dual role, oncogene, PDAC, tumor suppressor

## Abstract

Pancreatic ductal adenocarcinoma (PDAC) is characterized by its intricate biology governed by spatiotemporal dynamics in the expression and function of specific proteins. Here, DKK3 is identified as a dynamic player with a dual role in PDAC. Using the KRAS^G12D^‐driven mouse model with homozygous (DDKC) and heterozygous (DKC) DKK3 knockout, its stage and compartment‐specific functions are investigated. Knockout mice exhibited shorter lifespans with a higher incidence of high‐grade, desmoplastic, and metastatic cancers. DKK3‐deficient acini exhibited a marked increase in acinar‐to‐ductal metaplasia, with increased MAPK signaling and induction of the downstream effector *Fos*. During the progression of mouse and human PDAC, DKK3 expression shifted from epithelial dysplastic cells to cancer‐associated fibroblasts (CAFs). At the endpoint, DKK3‐expressing CAFs emerged as crucial contributors to tumor aggressiveness and fibrosis. Orthotopic transplantations confirm a stromal role, particularly in DDKC tumors, while mechanistic studies demonstrate that DKK3 activates IL6‐JAK‐STAT3 signaling and pro‐migratory/mesenchymal programs that are reversed by pharmacologic STAT3 inhibition in DDKC cells. Concordantly, *DKK3* expression correlates with IL6‐JAK‐STAT3 gene signatures in human PDAC datasets. Together, these findings underscore the intricate and context‐sensitive role of DKK3, delaying oncogenesis during early stages while paradoxically promoting tumor progression in later stages, suggesting that therapeutic targeting strategies should be approached with caution.

## Graphical abstract

In pancreatic cancer, the protein DKK3 is found to play a complex role: it initially slows early tumor growth but later promotes tumor aggressiveness. DKK3 expression evolves during progression and drives cancer severity by orchestrating cellular and molecular signaling pathways. Therefore, any attempt to therapeutically target DKK3 will need to be carefully considered.

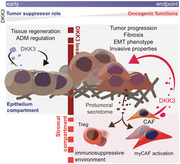

## 1 Introduction

Pancreatic ductal adenocarcinoma (PDAC) remains a persistent clinical challenge with a dismal prognosis due to therapy resistance and extensive tumor heterogeneity. Despite the identification of several biomarkers, the detection of PDAC remains inadequate, contributing to its low 5‐year survival rate of just 13%, one of the lowest among cancers.^[^ ^1^, ^2^, ^3^, ^4^ ^]^ The inefficacy of PDAC‐tailored chemotherapeutic treatment can be attributed not only to intra‐ and intertumoral heterogeneity but also to spontaneous and drug‐induced tumor evolution. At the cellular level, tumor intrinsic heterogeneity is driven by complex genetic, epigenetic, and protein alterations that fuel phenotypic selection in response to environmental pressures, providing tumors with remarkable adaptability.^[^ ^5^ ^]^ As such, the investigation of proteins impacting the intratumoral interplay between distinct cellular compartments in PDAC becomes of paramount importance.

Developmental pathways such as Wnt, Nodal/TGFβ, and Hedgehog signaling play critical roles in cancer initiation, maintenance, and progression, as well as in therapy‐driven heterogeneity and evolution.^[^ ^6^, ^7^, ^8^, ^9^ ^]^ Interestingly, numerous developmental genes that function as secreted proteins demonstrate a dual role in carcinogenesis. For example, the Hedgehog pathway acts as an oncogene in the early phases of tumor development but switches into a tumor suppressor as the cancer advances.^[^ ^10^ ^]^ Similarly, activin signaling functions as a tumor suppressor during the initial stages of tumor formation, only to promote tumor progression and metastasis in the later stages.^[^ ^11^ ^]^ In this context, we conducted a somatic‐to‐pluripotent stem cell reprogramming screen utilizing an shRNA library that targets 700 cancer‐related developmental genes. This approach aimed to uncover potential connections between tumor‐associated genes and embryonic development. Among the various targets identified, Dickkopf‐3 (DKK3), a secreted protein known for its diverse roles in cancer, emerged as a critical factor that restricts somatic cell reprogramming.^[^ ^12^ ^]^ DKK3, part of the Dickkopf protein family, acts through paracrine and autocrine mechanisms. For instance, during pancreas and liver regeneration, secreted DKK3 hinders recovery following injury,^[^ ^12^ ^]^ while knockout mice are viable and fertile, showing no noticeable phenotypic changes.^[^ ^13^ ^]^ This indicates that DKK3 has complex roles exhibiting both tumor‐suppressive and oncogenic functions in different cancer contexts.

Several studies have identified DKK3 also as a tumor suppressor,^[^ ^14^, ^15^, ^16^ ^]^ whereas others have characterized it as oncogenic.^[^ ^17^, ^18^ ^]^ In PDAC, previous studies have demonstrated that suppressing stromal DKK3 in a P53‐deficient context hampers tumor progression and prolongs survival in a preclinical trial format using DKK3‐targeting antibodies.^[^ ^19^ ^]^ Furthermore, DKK3‐mediated activation of YAP/TAZ in cancer‐associated fibroblasts (CAFs) has been associated with aggressive phenotypes in breast, colorectal, and ovarian cancers.^[^ ^17^ ^]^ These findings suggest that the oncogenic effects of DKK3 primarily arise from the tumor microenvironment (TME). However, our understanding of the role of epithelial DKK3 and its potential dynamics in the context of pancreatic carcinogenesis remains limited and unclear.

In this study, we elucidate the complex dual role of DKK3 in PDAC, showing that epithelial DKK3 acts as a tumor suppressor in early stages while stromal DKK3 functions as an oncogene in later stages. We also identified a previously unrecognized role of DKK3 in maintaining acinar integrity through the MEK‐Fos signaling axis, which contributes to delaying tumor progression. Notably, the early absence of DKK3 alters tumor biology and therapeutic responsiveness to STAT3 inhibition, highlighting its transition from a tumor suppressor to an oncogene. Our findings highlight DKK3's dual role in the progression of PDAC, revealing stage‐specific opportunities for intervention while also highlighting the need for caution in therapeutic approaches.

## 2 Results

### 2.1 DKK3 Exerts a Cell‐Autonomous Tumor Suppressor Role in Pancreatic Cancer Cells

**Figure 1:**
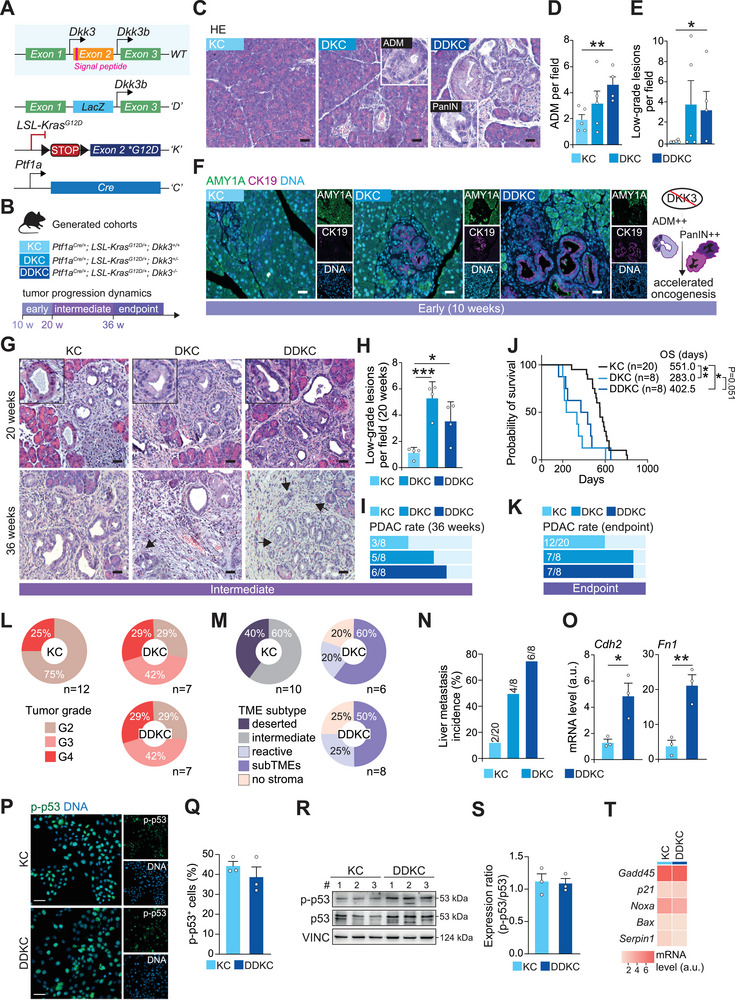
DKK3 exerts a cell‐autonomous tumor suppressor role in pancreatic cancer cells. A) Schematic representation of *Ptf1* ^Cre^ C), *LSL‐Kras^G12D^* (K), *Dkk3* wild‐type (WT), and knockout D) alleles. B) Overview of the different timepoints and mouse model genotypes. (C) Hematoxylin‐eosin (HE) staining on histological sections at the early time point (10 weeks). Inlets show higher magnification of ADM and PanIN lesions. Scale bar, 50 µm. D,E) Quantification of ADM (D) and low‐grade precursor lesions (E) in pancreata at the early time point. F) Immunofluorescence staining of pancreatic sections at early time point with AMY2A (green) and CK19 (purple). Scale bar, 50 µm. The right panel illustrates the main functions of DKK3 loss in accelerating tumorigenesis. G) HE staining on histologic sections of pancreata at intermediate (20, 36 weeks) time points. Inlets show higher magnification of ADM in KC and low‐grade precursor lesions in DKC and DDKC cohorts. White arrows indicate invading cells at 36 weeks. Scale bar, 50 µm. H) Quantification of low‐grade lesions in the pancreata at the intermediate time point (20 weeks). I) Incidence of invasive PDAC at 36 weeks. J) Kaplan‐Meier analysis of survival of KC, DKC, and DDKC mice. (H) Incidence of invasive PDAC in endpoint mice. L) Proportions of pathological tumor grading in endpoint cancers. M) Frequencies of TME phenotype in endpoint pancreata. N) Incidence of liver metastasis in endpoint mice. O) qRT‐PCR analysis of mesenchymal marker gene expression in tumor cells. P) Immunofluorescence staining for p‐S15 p53 (p‐p53; green) in tumor cells. Scale bar, 40 µm. Q) Quantification of the percentage of p‐p53^+^ area per field in KC and DDKC tumor cells. R,S) Western blot analysis (R) and quantification (S) of p‐p53 in KC and DDKC tumor cells. T) qRT‐PCR analysis of p53 downstream targets in KC and DDKC tumor cells. Data are means ± SEM. Each dot represents a mouse (D, E, H) or a cell line (O, Q, S). Significance was calculated by an unpaired Student's t‐test. Survival significance was calculated by the log‐rank (Mantel‐Cox) test. ^*^ *p* < 0.05; ^**^ *p* < 0.01; ^***^ *p* < 0.001. ADM, acinar‐to‐ductal metaplasia; OS, overall survival; PanIN, pancreatic intraepithelial neoplasia; PDAC, pancreatic ductal adenocarcinoma; TME, tumor microenvironment.

DKK3 was identified as a key regulator of multiorgan regeneration, specifically in the recovery from acute pancreatitis.^[^ ^12^ ^]^ To investigate the role of DKK3 during pancreatic tumorigenesis, we crossed a ubiquitous *Dkk3* knockout allele (D) into *LSL‐Kras^G12D/+^* (K); *Ptf1a^Cre/+^* (C) mice (KC), to generate homozygous DDKC and heterozygous DKC mouse models (**Figure** **1**A). To capture the dynamics of tumor progression, we conducted a comprehensive phenotypic characterization of the mouse models at various time points: starting from 10 weeks for the early stage, to intermediate stages at 20 and 36 weeks, and finally at the ethical endpoint (Figure 1B). At 10 weeks, the extent of acinar‐to‐ductal metaplasia (ADM) was greater in DKC and DDKC pancreata. In line, these mice already exhibited enlarged regions containing low‐grade pancreatic intraepithelial neoplasia (PanIN) lesions, whereas those were only rarely found in their age‐matched KC counterparts, suggesting a tumor suppressor role of DKK3 at the initiation stage (Figure 1C–F). At later time points, both low and high‐grade PanINs were more frequently found in DKC and DDKC mice by 20 weeks compared to the KC controls. The early formation of precursor lesions aligned with the faster development of invasive PDAC by 36 weeks in the knockout groups (DKC and DDKC), as opposed to KC mice (Figure 1G–I; Figure S1A–C, Supporting Information). Finally, the loss of DKK3 was associated with a significantly shortened survival in both DKC and DDKC, compared to KC controls (DDKC, 402.5 days versus DKC, 283.0 days versus KC, 551.0 days) (Figure 1J). Intriguingly, DKC mice exhibited a poorer overall survival median compared to both KC and DDKC mice, indicating that the single remaining *Dkk3* allele present in heterozygous DKC mice may still impact mouse survival (Figure 1J). At the endpoint, the DKC and DDKC mice showed an increased incidence of invasive PDACs with undifferentiated features and higher tumor grading (grade 3 and 4) compared to the KCs, which predominantly displayed a more differentiated phenotype (Figure 1K,L; Figure S1D, Supporting Information).

Intratumoral heterogeneity is a key hallmark of PDAC that significantly contributes to its malignant biology. Within spatially confined tumor regions, histologically and functionally distinct sub‐tumor microenvironments (subTMEs) – categorized as reactive, deserted, and intermediate – have been identified, their intratumoral co‐occurrence being associated with poor prognosis compared to uniform microenvironmental states.^[^ ^20^ ^]^ The analysis of endpoint cohorts revealed a higher incidence of co‐occurring subTMEs (≥ 2 distinct TMEs) in DKC and DDKC mice, corroborating their more aggressive phenotype, strongly contrasting with the KC mice, which exhibited either deserted or intermediate TME states (Figure 1M; Figure S1E, Supporting Information). Concomitantly, liver metastasis incidence was higher in DKK3‐knockout groups than in controls (Figure 1N; Figure S1D, Supporting Information).

Consistent with this invasive phenotype, isolated DDKC malignant cells, lacking DKK3 expression (Figure S1F, Supporting Information), exhibited increased intrinsic migration together with upregulated mesenchymal markers (*Cdh2*, *Fn1*) and, conversely, reduced expression of the epithelial adhesion molecule CDH1 (Figure 1O; Figure S1G–L, Supporting Information). By contrast, DDKC and KC lines showed comparable proliferation rates (Figure S1M, Supporting Information). To exclude involvement of a major tumor suppressor in mediating DKK3‐associated effects, we assessed p53 activity in tumor cells: neither p53 phosphorylation nor the expression of its downstream targets changed during tumor progression in KC or DDKC models, arguing against p53 loss (Figure 1P–T; Figure S1N, Supporting Information). Together, these data support a stage‐dependent role of DKK3, tumor‐suppressive in early disease yet potentially oncogenic in advanced PDAC, as reflected by the opposing survival outcomes in DKC vs DDKC mice.

### 2.2 DKK3 Safeguards Acinar Integrity and Restrains Dysplasia During PDAC Onset

To test this hypothesis, and building on our in vivo observation that DKK3 loss accelerates KRAS^G12D^‐driven dysplastic transformation, we next delineated DKK3's specific contribution to ADM, an early initiating event in PDAC. Our prior work showed that DKK3 governs acinar regeneration, marked by reactivation of embryonic programs, ADM, and incipient fibrosis, features characteristic of early pancreatic tumorigenesis, prompting us to interrogate DKK3's unique role in PDAC initiation.^[^ ^12^ ^]^

**Figure 2:**
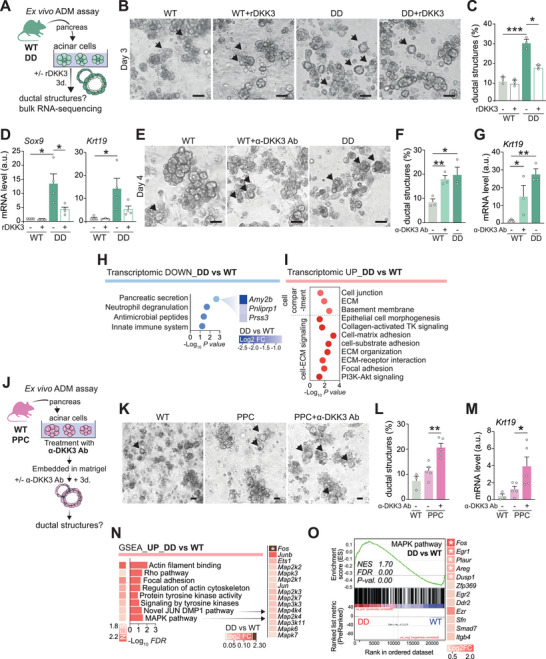
DKK3 safeguards acinar integrity and restrains dysplasia during PDAC onset. A) Experimental setup for ex vivo ADM assay B–E). (B and C) Brightfield images (B) and quantification of ductal structures (C) in acinar cultures treated or not with rDKK3 for three days. Arrows show ductal structures. Scale bar, 50 µm. (D) qRT‐PCR analysis of ductal marker gene expression in (A to C). (E and F) Brightfield images (E) and quantification of ductal structures F) in WT treated or not with neutralizing anti‐DKK3 antibody and DD acinar cultures after four days. Arrows show ductal structures. Scale bar, 50 µm. G) qRT‐PCR analysis of ductal marker gene *Krt19* expression in (E and F). H,I) DAVID‐based correlation analysis for down (H) and upregulated (I) genes of an RNA‐sequencing performed on acinar cultures after three days of ADM assay. J) Experimental setup for ex vivo ADM assay using neutralizing anti‐DKK3 antibody. K,L) Brightfield images (K) and quantification of ductal structures (L) in PPC (treated or not with neutralizing anti‐DKK3 antibody) and WT acinar cultures as in (J). Arrows show ductal structures. Scale bar, 50 µm. M) qRT‐PCR analysis of ductal marker gene *Krt19* expression in PPC (treated or not with neutralizing anti‐DKK3 antibody) and WT acinar cultures as in (J). N) GSEA of the transcriptomics data showing upregulated pathways with *FDR* ≤ 0.05 in DD versus WT acinar cultures. Heatmap represents log_2_ FC of top genes within the indicated gene sets. O) GSEA of the MAPK gene set in DD versus WT acinar cultures. Heatmap represents log_2_ FC of top genes within the indicated gene sets. Data are means ± SEM. Each dot represents a mouse (C,D,F,G,L,M). Significance was calculated by an unpaired Student's t‐test. ^*^ *p* < 0.05; ^**^ *p* < 0.01. ADM, acinar‐to‐ductal metaplasia; FC, fold change; FDR, false discovery rate; NES, normalized enrichment score.

Using an ex vivo acinar explant assay, we quantified ADM and transdifferentiation into duct‐like cells (**Figure** **2**A). Even on a wild‐type Kras background, acini from *Dkk3*‐null (DD) pancreata underwent rapid ADM, yielding significantly more ductal structures after three days in culture than wild‐type (WT; *Dkk3^+/+^*) counterparts (Figure 2B,C; Figure S2A, Supporting Information). Consistently, DD acini showed higher expression of ductal identity genes *Krt19* and *Sox9* (Figure 2D). To test whether DKK3 is induced during ADM, we measured its mRNA in WT cultures and observed a marked upregulation accompanying the morphological transition, consistent with a protective role of DKK3 in maintaining the acinar state (Figure S2B, Supporting Information). Functionally, exogenous recombinant DKK3 (rDKK3) reduced the emergence of ductal structures in DD cultures, rescuing the phenotype (Figure 2B–D; Figure S2A, Supporting Information). Conversely, neutralizing DKK3 by a blocking antibody (α‐DKK3 Ab) in WT acini recapitulated the DD phenotype, significantly increasing ductal structures relative to untreated controls (Figure 2E–G; Figure S2C, Supporting Information).

To capture global transcriptomic changes, we performed bulk RNA‐seq on WT and DD acinar cells after three days in culture. Loss of DKK3 was associated with diminished acinar functions, including enzyme secretion, and downregulation of gene sets related to ribosomal structure and mitochondrial translation. Given that pancreatic acinar cells have the highest ribosome.^[^ ^21^ ^]^ and protein content.^[^ ^22^ ^]^ among mammalian somatic cells, suppression of these pathways further supports compromised acinar integrity in DD acini (Figure 2H; Figure S2E, Supporting Information). Conversely, gene programs linked to epithelial morphogenesis and cytoskeletal remodeling were upregulated in DD acini, consistent with the pronounced ADM phenotype (Figure 2I). RNA‐seq showed similar overall *Dkk3* transcript levels in WT and DD acini, reflecting expression of the alternative intracellular isoform *Dkk3b* from a secondary transcriptional start site (Figure S2E, Supporting Information). Using exon‐specific primers, we confirmed loss of exon 2–containing transcripts encoding secreted full‐length DKK3, validating knockout of canonical DKK3, while *Dkk3b* expression remained unchanged in both genotypes (Figure S2F, Supporting Information).

To define the cell‐autonomous contribution of DKK3 loss during KRAS‐driven initiation, we repeated the ex vivo acinar explant assay using acini from KC and DDKC pancreata at 10 weeks (Figure S2G, Supporting Information). Mirroring the in vivo findings (Figure 1C–F), DDKC acini displayed significantly greater and accelerated ADM than KC after just one day in culture (Figure S2H,I, Supporting Information), accompanied by higher expression of ductal genes in DDKC and a time‐dependent induction of *Dkk3* in KC from day 1 to day 3 (Figure S2J, Supporting Information). By day 3, ADM in DDKC acini plateaued and no longer exceeded KC (Figure S2K,L, Supporting Information), consistent with oncogenic KRAS being the principal driver of ADM in the KC model. Thus, DKK3 loss accelerates the earliest step of tumor initiation, whereas DKK3 induction can delay, but not abolish, KRAS‐mediated ADM, underscoring KRAS dominance. Collectively, these data position DKK3 as a kinetic modulator rather than an absolute barrier to KRAS‐driven ADM.

Previous work by Zhou et al. in a KPC floxed background reported an oncogenic role for DKK3.^[^ ^19^ ^]^ Whether DKK3's tumor‐suppressive function operates independently of p53, however, remained unresolved. We first performed GSEA on differentially expressed genes between wild‐type and *Dkk3*‐null acinar cells and found no prominent changes in the p53 tumor‐suppressor pathway, arguing against p53 involvement at early stages (Figure S2M,N, Supporting Information). To directly test p53 independence, we used *Trp53^fl/fl^; Ptf1a^Cre/+^* (PPC) acini and carried out an ex vivo ADM assay with DKK3‐neutralizing antibodies (Figure 2J). Notably, DKK3 blockade markedly increased ductal structures relative to untreated controls, demonstrating that DKK3's tumor‐suppressive role persists in the absence of p53 (Figure 2K–M).

Altogether, these results underscore DKK3 role in preserving pancreatic acinar integrity and emphasize the tumor‐promoting potential of DKK3 loss during the earliest stages of pancreatic carcinogenesis.

### 2.3 Fos Orchestrates Tumor‐Promoting Effects of DKK3 Loss in Acinar Cells

**Figure 3:**
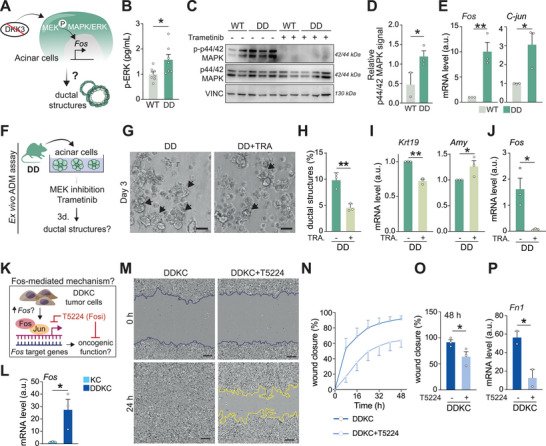
Fos orchestrates the tumor‐promoting effects of DKK3 loss. A) Proposed DKK3 loss‐mediated molecular mechanism driving ductal structure formation. B) ELISA for p‐ERK (p‐T202/Y204, p‐T185/Y187) on protein lysates from WT and DD acinar cultures after 3 days of ADM assay. C,D) Western blot analysis C) and quantification D) of p44/42 MAPK phosphorylation (p‐T202/Y204, p‐T185/Y187) levels in WT and DD acinar cells. E) qRT‐PCR analysis of *Fos* and *C‐jun* in WT and DD acinar cells. F) Experimental setup shown in G–J). (G and H) Brightfield images (G) and quantification of ductal structures (H) in DD acinar cultures treated or not with the MEK1/2 inhibitor trametinib for three days. Arrows show ductal structures. Scale bar, 50 µm. (I and J) qRT‐PCR analysis of ductal and acinar marker gene expression (I) and of *Fos* expression (J) in DD acinar cells treated or not with trametinib after three days. K) Experimental setup shown in L–P). (L) qRT‐PCR analysis of *Fos* expression in tumor cells. (M) Incucyte‐based wound healing assay on DDKC tumor cells treated or not with the transcription factor c‐Fos/AP‐1 inhibitor T5224, 48 h post‐wound generation. Scale bar, 25 µm. (N and O) Wound closure of DDKC tumor cells treated or not with T5224 at different time points (N) and at 48 h (O). (P) qRT‐PCR analysis of *Fn1* expression in DDKC tumor cells treated or not with T5224 for 72 h. Data are means ± SEM. Each dot represents a mouse (B,D,E,H–J) or a cell line (L,O,P). Significance was calculated by an unpaired Student's t‐test. ^*^ *p* < 0.05; ^**^ *p* < 0.01; ^***^ *p* < 0.001. ADM, acinar‐to‐ductal metaplasia; TRA, trametinib.

To probe mechanisms underlying DKK3's tumor‐suppressive role in acinar cells, we performed pre‐ranked GSEA on the DD versus WT RNA‐seq data, which highlighted MAPK, JUN, and DMP1 pathways, with *Fos* emerging as a key regulator (Figure 2N). Focused GSEA of the MAPK set identified *Fos*, *Egr1*, *Areg*, *Plaur*, and *Dusp1* among the top upregulated genes (Figure 2O). Consistent with these transcriptomic data, DD acini exhibited increased phosphorylation of p44/42 MAPK and induction of downstream targets Fos and Jun relative to WT cells (**Figure** **3**A–E; Figure S3A,B, Supporting Information). Despite robust mRNA induction, protein changes were modest; FOS showed only slight upregulation and JUN was unchanged, consistent with known post‐transcriptional and post‐translational control of AP‐1 components (e.g., differences in translation efficiency, protein half‐life, and stimulus‐dependent c‐Fos degradation.^[^ ^23^, ^24^ ^]^). Together, these findings support a model in which loss of DKK3 activates MEK/MAPK signaling to drive Fos‐dependent programs that accelerate ADM.

To substantiate the causal link between MAPK signaling and ADM formation upon DKK3 loss, we applied clinical‐grade MEK inhibitor trametinib on *Dkk3*‐null acinar cultures. Remarkably, MEK inhibition significantly reduced ductal structure formation in trametinib‐treated DD acini (Figure 3C,F,G,H). This decrease was accompanied by lower levels of the ductal marker *Krt19* and concomitant higher levels in the acinar marker *Amy1a* in treated *Dkk3*‐null cultures (Figure 3I). Importantly, a significant depletion in *Fos* mRNA levels was observed upon trametinib treatment, further supporting our hypothesis that Fos acts downstream of MAPK signaling to mediate ADM formation ex vivo (Figure 3J). In contrast, MEK inhibition in wild‐type acinar cells induced no significant changes in ductal structures or marker gene expression (Figure S3C–E, Supporting Information), reinforcing the involvement of MAPK cascade in *Dkk3* loss‐driven tumor initiation. In *Dkk3^+/+^* WT acinar cells, *Fos* is virtually undetectable at baseline and does not change upon MEK inhibition, indicating a low MEK‐FOS drive in the presence of DKK3 (Figure S3E, Supporting Information). Conversely, neutralizing DKK3 with the previously used blocking antibody (Figure 2) increased *Fos* mRNA in WT acini (Figure S3F, Supporting Information), though to a lesser extent than genetic DKK3 ablation.

Collectively, these results indicate that the tumor‐initiating functions promoted by DKK3 loss in early acinar cell transformation are governed by the activation of MAPK signaling pathway and subsequently mediated by the transcription factor Fos.

### 2.4 Fos Expression in DKK3‐Null Cancer Cells Mediates Invasive Properties

Next, we aimed to dissect the molecular alterations mediating the tumorigenic effects of DKK3 loss at end‐stage cancers. We therefore interrogated the status of the previously identified MAPK signaling cascade in cancer cells (Figure 3K–P; Figure S3G–M, Supporting Information). No significant difference in p44/42 MAPK phosphorylation was observed in DDKC cells compared to KC controls (Figure S3H,I, Supporting Information). To further corroborate this result, we assessed the tumor cell viability upon treatment with the MEK inhibitor trametinib. Again, no genotype‐specific sensitizing effect was induced upon MEK inhibition, indicating that this pathway lost its genotype‐specific relevance upon cancer progression in the absence of DKK3 (Figure S3J, Supporting Information). Interestingly, significantly higher levels of *Fos* – the previously identified downstream target of MAPK signaling – were found in DDKC cells, suggesting its potential role as a crucial driver of DKK3‐loss effects throughout tumor progression even in the absence of upstream MAPK signaling (Figure 3L). To substantiate the potential functional role of Fos at PDAC late stage, we conducted proliferation and wound healing assays using DDKC cells treated with the clinical‐grade selective Fos/AP‐1 inhibitor T5224, which prevents DNA‐binding and transactivation activities of the Fos/Jun complex. After 48 h, the migratory abilities of DDKC tumor cells were significantly abolished by the T5224 treatment, implying a concomitant reduction of their mesenchymal traits and oncogenic properties (Figure 3M–O). In line, qPCR analyses revealed significant downregulation of the Fos downstream target genes *Mmp2* and *Mmp3*, as well as of the mesenchymal marker *Fn1*, induced by the interference into Fos downstream effects (Figure 3P; Figure S3K, Supporting Information). Correspondingly, T5224 remained ineffective during wound healing assays in KC cells (Figure S3L, Supporting Information). Of note, cell viability remained unchanged upon inhibition of Fos binding in both genotypes (Figure S3M, Supporting Information). Altogether, these results show that loss of DKK3 leads to an upregulation of *Fos* expression, thereby mediating tumor aggressiveness and mesenchymal characteristics.

### 2.5 Epithelial DKK3 Inactivation Sustains an Aggressive Phenotype by Reprogramming CAFs and Immune Cells in the PDAC Microenvironment

Collectively, our data show that DKK3 acts cell‐autonomously to preserve acinar integrity in a KRAS wild‐type context and exerts a tumor‐suppressive role during KRAS^G12D^‐driven PDAC progression (Figures 1, 2, 3). Consistent with these roles, pancreatic tumors arising in heterozygous (DKC) and homozygous (DDKC) *Dkk3*‐deficient backgrounds display distinct histopathological features and TME landscapes. Notably, DKC mice exhibited even shorter survival than DDKC mice, despite loss of only a single *Dkk3* allele in both epithelial and stromal compartments in our non‐conditional *Dkk3*‐deficient model crossed with pancreas‐specific oncogenic KRAS, indicating a non‐linear, dose‐sensitive effect of DKK3 across various tissue compartments (Figure 1J).

**Figure 4:**
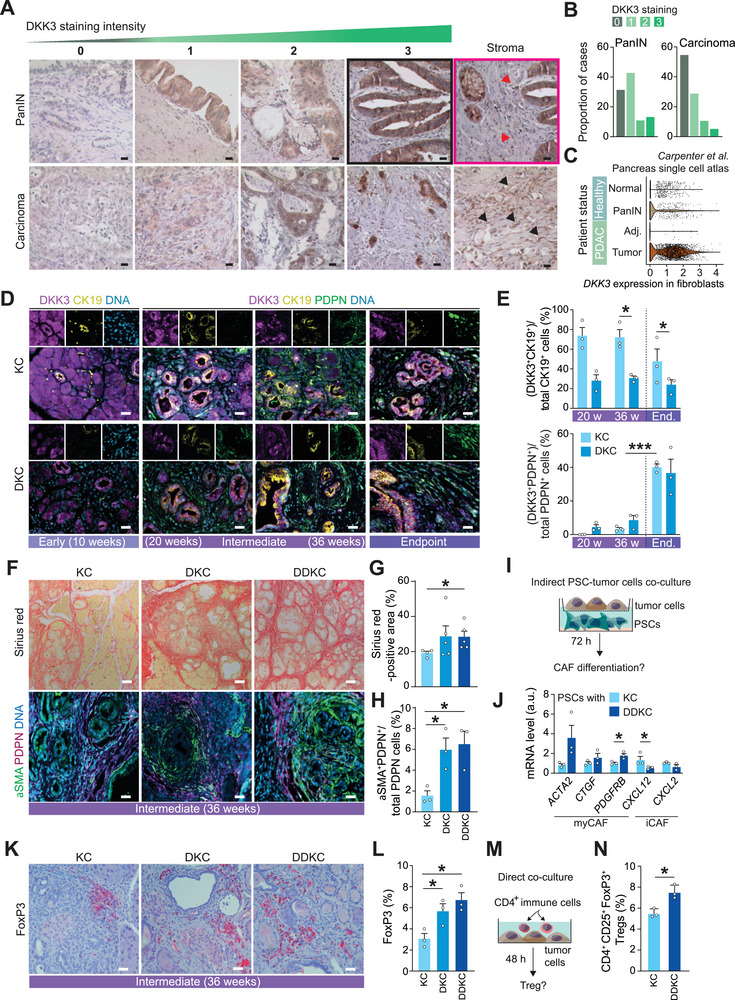
DKK3‐null tumors maintain their aggressive phenotype by reshaping the tumor microenvironment. A) Immunohistochemistry staining for DKK3 on sections of a human PDAC tissue microarray. Scale bar, 20 µm. Red arrows indicate PanIN‐associated fibroblasts, and black arrows represent CAFs. The “Stroma” panel in the PanIN row shows a crop from the same histological section as panel “PanIN 3*"*, reframed to emphasize the periductal stroma. Black and pink boxes represent color‐coded regions as shown in Figure S4B (Supporting Information) (source image). B) Proportion of PDAC cases with varying DKK3 expression levels in PanIN and carcinoma regions. C) Violin plot showing DKK3 expression in fibroblasts in normal and PanIN regions of healthy donors and tumor and adjacent normal tissues of PDAC patients. D,E) Immunofluorescence for DKK3 (purple), CK19 (yellow), and PDPN (green) on mouse pancreatic sections at early, intermediate, and end time points (D) (left panel) and quantification of DKK3^+^CK19^+^ and DKK3^+^PDPN^+^ populations (E) (right panels). Scale bar, 50 µm. F) Picrosirius red staining and immunofluorescence for αSMA (green) and PDPN (purple) on pancreatic sections at the intermediate time point. Scale bar, 50 µm. G,H) Quantification of picrosirius red‐positive (F) and αSMA^+^PDPN^+^ (G) areas (relative to total PDPN^+^ surfaces) in pancreatic tumors. I) Experimental design shown in J). (J) qRT‐PCR analysis of myCAF and iCAF marker gene expression in PSCs after co‐culture with tumor cells for 72 h. K) Immunohistochemistry staining for FoxP3 on pancreatic sections at the intermediate time point. Scale bar, 50 µm. L) Quantification of FoxP3^+^ areas in pancreatic tumors. M) Experimental setup shown in N). (N) Flow cytometry analysis of CD4^+^CD25^+^FoxP3^+^ Treg cell population after co‐culture of CD4^+^ T cells with tumor cells for 48 h. Data are means ± SEM. Each dot represents a mouse (E, G, H, L) or a cell line (J and N). Significance was calculated by an unpaired Student's t‐test. ^*^ *p* < 0.05; ^**^ *p* < 0.01; ^***^ *p* < 0.001. Adj., adjacent tissue; End., endpoint; PSC, pancreatic stellate cell.

Consequently, we sought to investigate the specific alterations in the TME induced by the loss of DKK3, which may contribute to the aggressive phenotype seen in DKC and DDKC tumors. To achieve this, we examined the intratumoral expression of DKK3 using a compartment‐specific approach. To evaluate global DKK3 expression in human PDAC tissues, we analyzed mRNA expression data from the publicly available TCGA PanCancer Atlas (n = 179).^[^ ^25^ ^]^ We observed a significantly higher *DKK3* expression level in PDACs compared to normal tissue, with no significant difference relative to the tumor grade (Figure S4A, Supporting Information). We then assessed DKK3 protein expression pattern by immunostaining on a human tissue microarray from a resected PDAC cohort.^[^ ^26^ ^]^ Whereas normal pancreatic tissue appeared mainly negative (score 0), different DKK3‐positive staining patterns (from weak to intense, score 1 to 3) were detected in PanINs and carcinomas. Specifically, 43.2% of patients (19 out of 44) displayed weak positivity (score 1) in PanIN precursor lesions, while 25.0% (11 out of 44) exhibited a moderate to intense expression pattern of DKK3 (scores 2 and 3) (**Figure** **4**A,B; Figure S4B, Supporting Information). Fourteen cases out of 44 (31.8%) exhibited no protein expression in PanINs. Also, low DKK3 expression was detected in PanIN‐associated fibroblasts (Figure 4A, right panels). Interestingly, this pattern changed when analyzing carcinoma‐containing regions of these patients. More than half of the PDACs (54.8%; 51 of 93) showed negative DKK3 staining in the epithelium, 30.0% (27 of 93) had a weak signal, and only 16.1% (15 of 93) displayed a moderate to intense DKK3 expression, irrespective of the tumor grade (Figure 4A,B; Figure S4C, Supporting Information). Intriguingly, intense DKK3 expression was detected in CAFs surrounding the tumor cells, which was only low expressed in PanIN‐associated fibroblasts (Figure 4A, right panels). These findings, coupled to our previous observations from our mouse model, suggest that, also in human PDACs, the early activation of DKK3 expression functions as a molecular tumor suppressive event during preneoplastic progression, which diminishes as malignant transformation occurs. In contrast, DKK3 expression in fibroblasts was found to be inversely regulated, as it was only detected in CAFs. To further substantiate these findings, we reanalyzed a publicly available single‐cell dataset from human PDAC patients.^[^ ^27^ ^]^ and investigated the expression of *Dkk3* across various cellular compartments. *Dkk3* expression was notably higher in fibroblasts compared to any other cell type and was nearly absent in malignant epithelial ductal cells (Figure S4D, Supporting Information), corroborating the compartment shift of DKK3 expression from epithelial toward fibroblastic cells upon PDAC progression. The re‐analysis of another publicly available pancreas single‐cell atlas, which includes samples from healthy donors, PDAC patients, and adjacent normal tissues, similarly revealed a notable increase in *DKK3* expression in fibroblasts transitioning from the PanIN stage to tumor stage (Figure 4C).

We thus examined the compartment‐specific expression of DKK3 in our mouse pancreatic tissues (KC, harboring two wild‐type *Dkk3* alleles versus DKC, carrying one wild‐type *Dkk3* allele) at various stages (early to late), using co‐stainings for the epithelial ductal marker CK19 and the pan‐CAF marker PDPN. At the early and intermediate stages, as ADM and low‐grade PanIN lesions developed, DKK3 expression was primarily localized in epithelial cells, including acinar cells undergoing ADM and preneoplastic ductal cells forming precursor lesions, in both KC and DKC genotypes, while its expression was totally absent in both compartments in DDKC pancreata (Figure 4D,E; Figure S4E–G, Supporting Information). During this phase, emerging PDPN^+^ fibroblasts surrounding these non‐invasive precancerous lesions showed only negligible DKK3 expression. Upon PDAC progression (endpoint), DKK3⁺PDPN⁺ CAFs increased in both KC and DKC tumors, predominating in DKC but remaining comparable to DKK3⁺CK19⁺ tumor cells in KC, indicating a shift of DKK3 expression from epithelial to stromal compartments (Figure 4D,E). Similar patterns were observed in the human PDAC TMA and in the re‐analysis of the publicly available pancreas single‐cell atlas (Figure 4C).

To further investigate the tumor‐promoting functions of DKK3, we specifically examined the stromal compartment's response to DKK3 loss. Picrosirius red staining was performed to visualize tumor fibrotic content, revealing considerably more pronounced fibrosis in both DKC and DDKC mice at the intermediate time point (Figure 4F,G). Myofibroblasts are known to play a crucial role in collagen production within the PDAC TME.^[^ ^28^ ^]^ To pinpoint the dominant CAF subtype in this fibrotic TME, we conducted staining for the myofibroblastic CAF marker αSMA. Our findings demonstrated a notable enrichment of PDPN^+^αSMA^+^ myCAFs in both DKC and DDKC tumors when compared to KC at the intermediate stage (Figure 4F,H). To further substantiate the capacity of *Dkk3*‐null tumor cells to independently reshape their TME toward a myCAF phenotype, we utilized a cell–cell contact‐free co‐culture system involving malignant cells and pancreatic stellate cells (PSCs) as CAF precursors,^[^ ^29^ ^]^ and traced their differentiation into distinct CAF subtypes (Figure 4I). Strikingly, *Dkk3*‐null cancer cells led to an increase in myCAF gene expression (*ACTA2*, *CTGF*, *PDGFRB*), with concomitant downregulation of inflammatory CAF markers (*CXCL12*, *CXCL2*) in PSCs (Figure 4J).

The identification of TME modulations, marked by an abundance of myCAFs, indicates that DKK3 loss‐of‐function in cancer cells may also impact the stromal immune landscape. When comparing the pancreata of KC mice to those with DKC and DDKC tumors, we observed a significant increase in CD3^+^ T lymphocyte populations at the intermediate time points of 20 and 36 weeks (Figure S4H, Supporting Information). Furthermore, analysis of the immunosuppressive regulatory T cell (Treg) subset, using specific FoxP3 immunostaining, revealed a higher prevalence of FoxP3^+^ T cells in DKK3‐knockout tumors (Figure 4K,L; Figure S4I, Supporting Information). Of note, CD4^+^ T cells serve as precursors to CD4^+^FoxP3^+^ Tregs.^[^ ^30^ ^]^ To examine the intrinsic capacity of *Dkk3*‐null tumor cells to redraft the T‐cell immune compartment, we employed direct co‐culture of tumor cells and CD4^+^ T cells (Figure 4M). After 48 h of direct co‐culture, DDKC tumor cells displayed a greater potential to differentiate activated CD4^+^ T cells into CD4^+^FoxP3^+^ Tregs than KC cells (Figure 4N). In line, we observed significantly lower CD8^+^ cytotoxic T cell content in DDKC tumors at 36 weeks (Figure S4J, Supporting Information), functionally corroborating the development of an immunosuppressive phenotype in DKK3‐knockout context.

Collectively, these findings suggest that the loss of DKK3 leads to TME reprogramming toward a fibrotic niche enriched with myCAFs and immunosuppressive Tregs, ultimately enhancing tumor aggressiveness.

### 2.6 CAF‐Derived DKK3 Fosters Aggressiveness of PDAC Late Stage

DKK3 functions as a tumor suppressor in early PDAC (Figures 1C–F and 2). Given its reported oncogenic roles in other cancers.^[^ ^17^, ^18^, ^19^ ^]^ and our observation of stage‐ and compartment‐specific DKK3 expression during PDAC progression (Figure 4A–E), we hypothesized a context‐dependent switch whereby DKK3 is tumor‐suppressive initially but becomes oncogenic at later stages.

**Figure 5:**
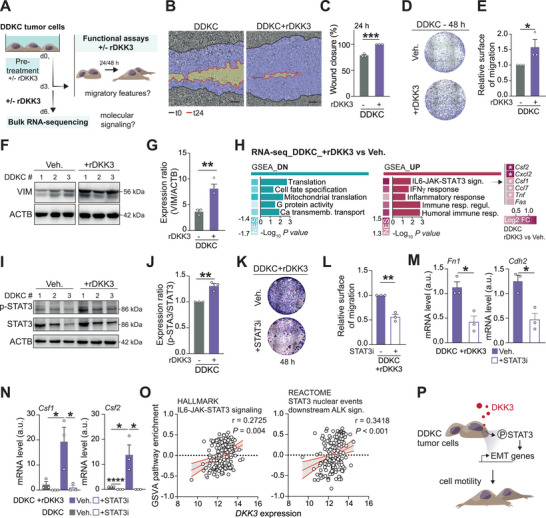
IL6‐JAK‐STAT3 signaling mediates the oncogenic activity of DKK3. A) Experimental setup of migration assays shown in B–F). (B) Incucyte‐based wound healing assay on DDKC tumor cells treated or not with rDKK3, 24 h post‐wound generation. Scale bar, 25 µm. (C) Wound closure of DDKC tumor cells treated or not with rDKK3 at 24 h. (D) Boyden chamber assay with DDKC tumor cells treated or not with rDKK3 for 48 h. (E) Relative surface of migration of DDKC tumor cells treated or not with rDKK3 for 48 h. F,G) Western blot analysis (F) and quantification (G) of VIM levels in DDKC tumor cells treated or not with rDKK3 for 72 h. H) GSEA of the transcriptomics data showing downregulated and upregulated gene sets with *P* value ≤ 0.05 in rDKK3‐treated DDKC versus DDKC tumor cells. Heatmap represents log_2_ FC of top genes within the indicated gene sets. I,J) Western blot analysis (I) and quantification (J) of p‐Y705 STAT3 (p‐STAT3) in DDKC cells, treated or not with rDKK3. K) Boyden chamber assay with rDKK3‐treated DDKC tumor cells, treated or not with the STAT3 inhibitor (STAT3i) stattic for 48 h. L) Relative surface of migration of rDKK3‐treated DDKC tumor cells, treated or not with STAT3i for 48 h. M) qRT‐PCR analysis of mesenchymal marker (*Fn1* and *Cdh2*) expression in rDKK3‐treated DDKC tumor cells, treated or not with STAT3i for 48 h. N) qRT‐PCR analysis of IL6‐JAK‐STAT3 target gene expression (*Csf1*, *Csf2*) in DDKC and rDKK3‐treated DDKC tumor cells, treated or not with STAT3i for 48 h. O) Correlation plot showing the correlation between *DKK3* expression and IL6‐JAK‐STAT3 signaling. P) Illustrative summary of the molecular mechanism underlying the oncogenic role of DKK3. Data are means ± SEM. Each dot represents a cell line (C,E,G,J, L–N). Significance was calculated by an unpaired Student's t‐test. ^*^ *p* < 0.05; ^**^ *p* < 0.01. DN, downregulated; FC, fold change; NES, normalized enrichment score; sign., signaling; UP, upregulated; Veh., vehicle.

To test this, we treated end‐stage *Dkk3*‐null tumor cells with stable mesenchymal features (Figure 1O) with rDKK3 (**Figure** **5**A). rDKK3 markedly enhanced cell migration and increased the mesenchymal marker vimentin, consistent with an oncogenic effect that mimics stromal DKK3 secretion (Figure 5B–G). Bulk RNA‐seq of untreated versus rDKK3‐treated DDKC cells (modeling CAF‐derived DKK3) identified IL6‐JAK‐STAT3 signaling as one of the most upregulated pathways (Figure 5H). Western blots confirmed pathway activation, showing increased STAT3 phosphorylation in rDKK3‐treated cells (Figure 5I–J). Functionally, pharmacologic STAT3 inhibition with stattic (Figure S5A–B, Supporting Information) reduced rDKK3‐induced migration and lowered expression of mesenchymal markers *Cdh2* and *Fn1* (Figure 5K–M), while having no effect on non‐stimulated DDKCs (Figure S5C–D, Supporting Information), indicating that STAT3 activity specifically mediates rDKK3's effects. RNA‐seq further nominated *Csf1*, *Csf2*, and *Cxcl2* as downstream targets (Figure 5H). qPCR validated rDKK3‐dependent induction of *Csf1* and *Csf2* and their suppression by stattic, with *Cxcl2* showing the same trend (Figure 5N; Figure S5E, Supporting Information). Analysis of TCGA datasets demonstrated a strong correlation between DKK3 expression and IL6‐JAK‐STAT3 signatures across multiple gene sets (Hallmark, Reactome) (Figure 5O). Together, these data establish IL6‐JAK‐STAT3 signaling as a key mediator of oncogenic DKK3 activity and highlight this pathway as a potential therapeutic target in advanced PDAC (Figure 5P).

**Figure 6:**
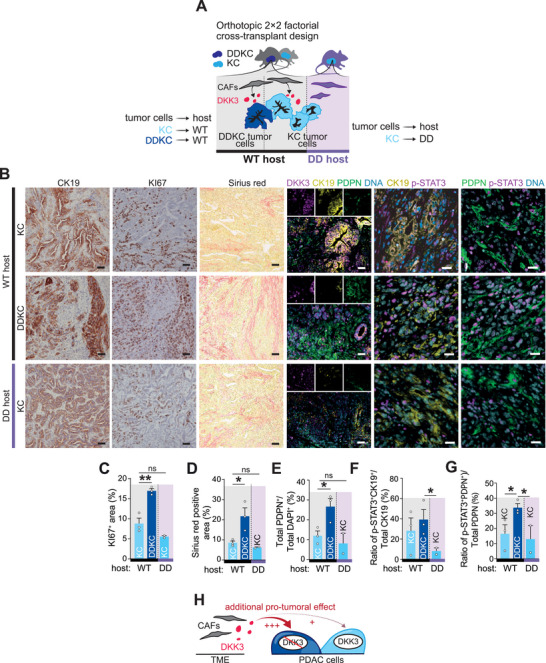
DKK3‐expressing fibroblasts act in an oncogenic manner at end‐stage PDAC. A) Experimental design of the orthotopic assay shown in B–G). (B) Immunohistochemistry staining for CK19 and KI67, picrosirius red staining, and immunofluorescence for DKK3 (purple), CK19 (yellow), and PDPN (green), and p‐STAT3 (purple), CK19 (yellow), and PDPN (green) on sections of resected tumors arising from KC and DDKC tumor cells transplanted in WT host, and KC tumor cells transplanted in DD host. Scale bar, 50 µm. (C and D) Quantification of KI67 (C) and picrosirius red (D) positive area in resected tumors is shown in (B). (E) Quantification of PDPN^+^ cells in resected tumors is shown in (B). (F) Quantification of p‐STAT3^+^CK19^+^ cells (relative to total CK19^+^ cells) in resected tumors is shown in (B). (G) Quantification of p‐STAT3^+^ PDPN^+^ cells (relative to total PDPN^+^ cells). H) Illustrative summary of the orthotopic assay (B to G). Data are means ± SEM. Each dot represents a mouse (C‐G). Significance was calculated by an unpaired Student's t‐test. ^*^ *p* < 0.05; ^**^ *P* < 0.01; ^***^ *P* < 0.001. CAFs, cancer‐associated fibroblasts; PDAC, pancreatic ductal adenocarcinoma; TME, tumor microenvironment.

To validate our findings in vivo and dissect tumor–stroma crosstalk by DKK3 status, we used an orthotopic 2 × 2 factorial cross‐transplant design: malignant cells from endpoint KC or DDKC tumors were orthotopically implanted into the pancreata of wild‐type or *Dkk3*‐null hosts, generating four groups (KC→WT, KC→DD, DDKC→WT, DDKC→DD) and isolating DKK3 contributions from tumor versus stroma (**Figure** **6**A; Figure S6A, Supporting Information). KC cells engrafted into WT or DD hosts formed well‐differentiated tumors (Figure 6B), recapitulating the autochthonous KC phenotype (Figure S1D, Supporting Information). By contrast, DDKC→WT tumors were undifferentiated (Figure 6B), supporting genotype‐specific behavior in this system. Notably, DDKC→WT tumors showed the highest proliferation index versus KC→WT or KC→DD, the most pronounced collagen deposition, and CAF enrichment in WT stroma receiving DDKC cells (Figure 6B–E). In contrast, DDKC→DD transplants phenocopied end‐stage lesions observed in the autochthonous model, although interpretation is limited by the survival of only a single mouse (Figure S6A, Supporting Information). The fraction of DKK3^+^PDPN^+^ CAFs was similar between KC and DDKC tumors in WT hosts (Figure 6B; Figure S6B, Supporting Information) and, as expected, absent in DD hosts (Figure 6B; Figure S6A, Supporting Information). A small DKK3^+^CK19^+^ epithelial population appeared in WT hosts engrafted with DDKC cells, likely host‐derived (Figure 6B; Figure S6C, Supporting Information). Consistent with in vitro data, p‐STAT3^+^CK19^+^ tumor cells were most abundant in DKK3‐expressing CAF contexts (Figure 6B,F; Figure S6A, Supporting Information). CAFs themselves also exhibited STAT3 activation (p‐STAT3^+^PDPN^+^), suggesting a paracrine, and potentially autocrine, component of stromal DKK3‐STAT3 signaling (Figure 6B,G).

In conclusion, our mouse model illustrated the dual role of DKK3 in the progression of PDAC. While DKK3 derived from CAFs in wild‐type pancreas enhances the oncogenic properties of cancer cells, its impact was notably more pronounced in DDKC tumor cells, which already displayed an aggressive mesenchymal phenotype (Figure 6H). This aggressive phenotype is initially driven by the cell‐autonomous pro‐tumorigenic effects resulting from the loss of DKK3 function.

## 3 Discussion

Due to significant intra‐ and intertumoral heterogeneity, coupled with challenges in early detection, PDAC remains one of the deadliest cancers, characterized by poor prognosis and low survival rates. This underscores the urgent need to identify key druggable targets that drive cancer aggressiveness and facilitate the oncogenic interplay between tumor and stroma. We have previously identified DKK3 as a barrier to multiorgan regeneration, including recovery from experimental pancreatitis, and as a biomarker in patients following pancreatic injuries, such as acute and chronic pancreatitis.^[^ ^12^ ^]^

**Figure 7:**
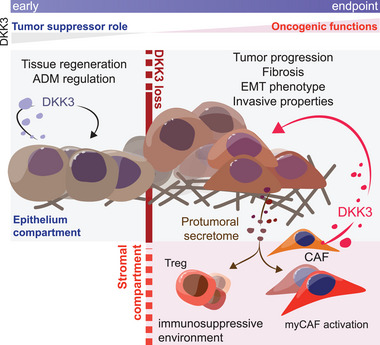
DKK3 functions in a stage‐specific manner during pancreatic cancer progression. Model recapitulating the dual role of DKK3 in pancreatic cancer. DKK3 preserves acinar integrity at early stages through MEK‐Fos signaling, thereby acting as a tumor suppressor. In contrast, at advanced stages, stromal DKK3 promotes tumor aggressiveness via IL6‐JAK‐STAT3 signaling, highlighting its context and stage‐dependent oncogenic role. ADM, acinar‐to‐ductal metaplasia; CAF, cancer‐associated fibroblast; myCAF, myofibroblastic CAF; Treg, regulatory T cell.

Recognizing that regeneration begins in the acinar compartment – which is characterized by the reactivation of embryonic signaling, ADM, and the onset of fibrosis, all of which coincide with the early stages of pancreatic cancer – we considered it necessary to investigate the role of DKK3 in the initiation of this disease. Notably, DKK3 has consistently been recognized as a tumor suppressor across various cancer types.^[^ ^16^, ^31^, ^32^ ^]^ Aberrant promoter methylation can frequently lead to the downregulation of DKK3, thereby exerting a tumor‐suppressive effect. While most studies investigated the role of DKK3 in established cancers, none of these specifically explored its role during cancer initiation. In PDAC, we found that DKK3 loss facilitates tumor‐initiating functions in acinar cells, promoting their transformation into ductal structures, even in a KRAS wild‐type context (**Figure** **7**). The RAF‐MEK‐ERK pathway is likely the most crucial signaling axis influencing PDAC biology and its progression.^[^ ^33^ ^]^ While the loss of DKK3 alone does not directly drive tumorigenesis,^[^ ^13^ ^]^ our data suggest that DKK3 loss cooperates with oncogenic KRAS to accelerate carcinogenesis. Here, we have identified ERK‐Fos signaling as a key player in the tumor‐initiating functions linked to DKK3. Notably, the ERK‐Fos axis has been shown to facilitate the progression from PanIN lesions to PDAC.^[^ ^34^ ^]^ Our research underscores DKK3 as a crucial factor in limiting the ERK signaling cascade and suppressing the immediate early gene *Fos*. Interestingly, the upregulation of *Fos* following DKK3 loss initially appears to depend on the MEK‐ERK pathway. However, once the progression to a cancerous state occurs, this upregulation seems to become independent of upstream regulatory factors. At advanced stages, the oncogenic function of DKK3 is instead mediated by IL6‐JAK‐STAT3 signaling, a well‐established driver of PDAC progression, promoting tumor cell proliferation, invasion, and resistance to therapy.^[^ ^35^, ^36^ ^]^ Targeting this pathway may therefore offer therapeutic potential for DKK3‐positive tumors. Analysis of publicly available TCGA data revealed an upregulation of *DKK3* in PDAC compared to normal pancreatic tissue. Furthermore, through staining for DKK3 using tissue microarrays and genetically engineered mouse models (GEMMs), we demonstrated elevated expression levels in acinar cells and PanINs in comparison to carcinoma. This further supports the notion of DKK3's early role as a tumor suppressor in the progression of pancreatic cancer.

At first glance, our evidence for an early tumor‐suppressive role of DKK3 seems at odds with prior work in a floxed KPC model, where *Dkk3* deletion reduced invasiveness and prolonged survival.^[^ ^19^ ^]^ That rapid, *Trp53*‐deficient setting likely obscured early, context‐dependent DKK3 functions. Using KC mice with heterozygous or homozygous *Dkk3* loss, we mapped DKK3 over time and captured a functional switch from early suppression to late oncogenic activity. Importantly, DKK3's early acinar‐protective effect is p53‐independent: in p53‐null, KRAS‐wild‐type acini, DKK3 neutralization significantly increased ADM. These findings reconcile the KPC result, where DKK3 loss ultimately extends survival, by placing the switch at later stages. To broaden relevance, DKK3 should be tested in additional aggressive PDAC models (e.g., *LSL‐Kras^G12D/+^*; *Ink4a/Arf^fl/fl^; Ptf1a^Cre/+^*) and in *TP53*‐mutant contexts, as >50% of human PDAC harbor *TP53* mutations.^[^ ^37^ ^]^ Our study deliberately interrogated early initiation under intact p53. Future work in p53‐mutant models will be essential to define the full spectrum of DKK3 activity.

Despite such an oncogenic role of DKK3 in non‐pancreatic cancers,^[^ ^14^, ^15^, ^17^, ^18^, ^19^ ^]^ we demonstrate that the role of DKK3 is more nuanced. For instance, in solid tumors, the stromal expression of DKK3 has been associated with increased cancer aggressiveness through its modulation of the protumorigenic effects of CAFs.^[^ ^17^ ^]^ More specifically, in pancreatic cancer, DKK3 derived from PSCs has been shown to promote tumor growth, dissemination, and chemoresistance.^[^ ^19^ ^]^ Our findings suggest that DKK3 exerts a complex dual role, functioning as a tumor suppressor in normal epithelial acinar cells while enhancing the tumorigenic potential of transformed cancer cells (Figure 7).

DKK3 has established roles in modulating various aspects of the TME, such as activation of CAFs and the immune modulation of CD8^+^ T cells.^[^ ^17^, ^38^, ^39^ ^]^ However, given that DKK3 is just one of many secreted factors in PDAC, the reprogramming of the TME observed in DDKC tumors likely arises not from stromal DKK3 but from the unique intrinsic features and the subsequently altered secretome of DKK3‐knockout cancer cells. Although fibroblasts are present during the intermediate stages of PDAC, only low DKK3 expression was detected in either the GEMM or the human TME surrounding PanIN lesions, which was further corroborated by the re‐analysis of a unique pancreas single cell atlas that includes PanIN‐bearing healthy tissues, PDAC, and adjacent normal tissues.^[^ ^40^ ^]^ This indicates that stromal DKK3 expression surrounding tumor cells increases after carcinoma develops, emphasizing the stage‐specific nature of DKK3, in line with a previous publication that identified DKK3 as one of the main proteins present in tumor‐proximal PDPN^+^ CAFs.^[^ ^41^ ^]^ In our allograft mouse studies, we demonstrated an oncogenic role for stromal DKK3, which significantly promoted tumor growth when paired with DKK3‐deficient tumor cells compared to those with proficient DKK3 expression. This contrast highlights the protective role of epithelial DKK3 in proficient KC cells. In the absence of DKK3, DDKC cells activated oncogenic programs, resulting in a more aggressive phenotype further enhanced by the presence of stromal DKK3 in vivo.

Additionally, the significantly reduced survival observed in heterozygous DKC mice compared to homozygous DDKC animals may be linked to the influence of stroma‐derived DKK3 on epithelial cells, which have already acquired oncogenic features due to the loss of one *Dkk3* allele. DKK3 expression is notably found in CAFs within human PDAC single‐cell datasets but is lost or downregulated in the carcinomas of PDAC patients. This trend was also seen in our orthotopic mouse model, where wild‐type hosts were transplanted with DDKC tumor cells. Further research is necessary to explore the molecular mechanisms underlying the oncogenic role of DKK3 in our system and to identify potential druggable targets.

The autochthonous *Dkk3* mouse model was created by replacing exon 2, which contains the signal sequence necessary for protein secretion.^[^ ^13^ ^]^ However, the role of DKK3 may be more complex; a recent study identified a second intracellular gene product, DKK3B, originating from the same *Dkk3* locus, but from a separate transcriptional start site located in intron 2.^[^ ^42^ ^]^ Knocking out the *Dkk3b* transcript while preserving secreted DKK3 resulted in embryonic lethality prior to implantation. Our research focused solely on the role of secreted DKK3 and did not investigate this second transcript; thus we cannot rule out the potential intracellular role of DKK3B in promoting protumoral programs.

Overall, our findings underscore the dual role of DKK3 in pancreatic cancer, illustrating its stage‐ and compartment‐specific expression that yields both tumor‐suppressive and oncogenic functions depending on the DKK3‐secreting cell type.

Given its context‐dependent functions, DKK3‐directed therapy should be stage‐ and biomarker‐guided. Preservation of DKK3 signaling may benefit preneoplastic/early acinar settings, but PDAC's late presentation limits this option. In established disease, where stromal DKK3 activates IL6‐JAK‐STAT3 and migratory programs, inhibition is the rational strategy. Candidates include tumors with high stromal DKK3 and STAT3‐enriched signatures, using p‐STAT3 as a pharmacodynamic readout. Combination with JAK/STAT inhibitors, and where MAPK‐AP‐1 activation is evident, MEK‐FOS antagonists may enhance efficacy, consistent with our data showing that STAT3 blockade suppresses DKK3‐driven migration and mesenchymal features. Safety considerations argue against use during pancreatitis/early acinar injury; biomarker‐enriched adjuvant windows and metastatic settings are plausible entry points. Preclinical data with the DKK3 antibody JM6‐6‐1 showing survival benefit support targeting the DKK3‐STAT3 axis in late‐stage, biomarker‐selected PDAC. Finally, because epithelial DKK3 is lost in ∼30% of PDAC, it may serve as a prognostic marker of rapid early progression, whereas high stromal DKK3 with STAT3 activation may identify patients for anti‐DKK3 strategies. Larger cohorts are needed to define clinical utility.

## 4 Experimental Section

### Study Design

The aim of the study was to decipher the role of DKK3 during the development and progression of PDAC by using GEMMs and various in vivo, ex vivo, and in vitro assays, as well as by re‐analyzing publicly available PDAC single cell‐resolved sequencing data sets. Accordingly, the objectives were achieved by (i) analyzing the functional role of DKK3 during KRAS^G12D^‐driven pancreatic tumorigenesis employing GEMMs, (ii) characterizing tumor‐initiating capacities and underlying molecular mechanisms behind DKK3 loss‐driven tumor onset using ADM ex vivo assays and in vitro experiments, and (iii) assessing the oncogenic role of DKK3 at PDAC late stage in a specialized orthotopic mouse model as well as with in vitro assays. DKK3 expression in human PDACs was assessed on a TMA and using published single‐cell sequencing data sets. For all mouse studies, sample sizes were defined by biostatisticians from the Institute for Epidemiology and Medical Biometry (University Hospital of Ulm); animals were randomly assigned to experimental groups. Analysis of mouse PDAC pathology and of the human PDAC TMA by board‐certified pathologists (T.F.E.B. and P.M., respectively) were performed in a blinded fashion. Study design was both comparative and iterative, with data from one experiment being used to inform the direction of subsequent investigations.

### Ethics Statement

All animal care and procedures followed German legal regulations and were approved by the governmental review board of the state of Baden‐Württemberg (permission #: 1313, 1590, O.195‐11, O.195‐19). All mouse work aspects were carried out following strict guidelines to ensure careful, consistent, and ethical handling of mice. The human patient material used in this study was provided by the Institute of Pathology of the University Hospital of Ulm following the regulations and the vote of the Ethics Committee of the University of Ulm (ethics #: 255/14).

### Mice

*Dkk3* knockout mice used in this study were obtained from Christof Niehrs.^[^ ^13^ ^]^ *Dkk3* ^+/+^, *Dkk3* ^+/−^, and *Dkk3* ^−/−^ mice were crossed with pancreas‐specific *Ptf1a^Cre^*.^[^ ^43^ ^]^ and *LSL‐Kras^G12D^*.^[^ ^44^ ^]^ to generate *LSL‐Kras^G12D/+^*; *Ptf1a^Cre/+^* (KC), *Dkk3* ^+/−^; *LSL‐Kras^G12D/+^*; *Ptf1a^Cre/+^* (DKC), and *Dkk3* ^−/−^; *LSL‐Kras^G12D/+^*; *Ptf1a^Cre/+^* (DDKC) mice. *Trp53^fl^*.^[^ ^45^ ^]^ mice were crossed with *Ptf1a^Cre^* animals to generate *Trp53^fl/fl^*; *Ptf1a^Cre/+^* (PPC). For the syngeneic orthotopic assay, 8‐week‐old *Dkk3* ^+/+^ and *Dkk3* ^−/−^ mice were used. Eight to 20‐week‐old *Dkk3* ^+/+^ mice were used for CD4^+^ T cell isolation. All the mice were housed and bred in a conventional health status‐controlled animal facility. All the aspects of the mouse work were carried out following strict guidelines to ensure careful, consistent, and ethical handling of mice.

### Cell Culture

KC and DDKC cell lines were respectively isolated from KC and DDKC mice and derived as described previously.^[^ ^46^ ^]^ Cells were cultured in DMEM, containing 10% FBS and P/S (100 IU mL^−1^ penicillin and 100 µg mL^−1^ streptomycin sulfate). Human PSCs^[^ ^29^ ^]^ were kindly provided by Matthias Löhr (Karolinska Institute) and were cultured in DMEM, containing 10% FBS and P/S. All cells were propagated at 37 °C under 5% (v/v) CO_2_ atmosphere. All experiments were performed between passages 5 and 25. All experiments were conducted with at least two different cell lines per genotype. Mycoplasma tests were regularly performed using the Mycoprobe mycoplasma detection kit (R&D Systems).

### Ex Vivo ADM Assay

Acini were isolated as previously described.^[^ ^47^ ^]^ Cells were Matrigel‐GFR (Corning)‐embedded in 24‐well plates. Once the Matrigel stiffened, Waymouth's medium containing 10% FBS, 1% PS, 0.1 mg mL^−1^ of trypsin inhibitor (ThermoFisher Scientific), and 10 µg mL^−1^ dexamethasone was applied, and cells were incubated for three days at 37 °C under 5% (v/v) CO_2_ atmosphere. Acinar cultures were treated with 10 µg mL^−1^ of rDKK3 (Sino Biological), with 0.025 ng mL^−1^ trametinib (Selleckchem), or with 40 µg mL^−1^ of monoclonal neutralizing anti‐DKK3 antibody (BioXCell). For subsequent RNA and protein isolation, cells were incubated in cell recovery solution (Corning) for 30 min on ice. Brightfield images were taken using an EVOS FL Auto Imaging System. Ductal structures were counted for at least ten independent pictures per condition and mouse. Proportions of ductal structures were evaluated relative to the total number of acini present per field. A limitation of the ex vivo ADM assays is the reliance on ECM components, which were known to have batch‐to‐batch variabilities. These variations can significantly influence ADM propensity, causing shifts in the absolute rates of ductal structure formation between experiments. Our interpretations were therefore based on comparisons within experiments rather than on absolute percentages across experiments.

### Co‐Culture Assay

For assessing CAF differentiation, PSCs were propagated on Matrigel GFR‐coated wells (1:4 dilution) in serum‐free DMEM containing P/S. For co‐culture approaches assessing PSC differentiation, tumor cells and PSCs (1:1 ratio; 50 000 tumor cells in 6‐well cell culture inserts (0.4 µm pore size ThinCert, Greiner Bio‐One) and 50 000 PSCs) were propagated in serum‐free DMEM containing P/S. All analyses to investigate PSC differentiation into CAFs were conducted using the vehicle‐inactivated PSC condition as a reference for normalization.

For the evaluation of T cell differentiation, splenocytes were isolated as previously described.^[^ ^48^ ^]^ and subsequent CD4^+^ T cell isolation was performed using CD4^+^ T Cell Isolation Kit (Miltenyi Biotec), following the manufacturer's protocol. Prior to the start of co‐culture, CD4^+^ T cells were activated for 24 h using Dynabeads Mouse T‐Activator CD3/CD28 (ThermoFisher Scientific). Direct co‐culture was performed in 6‐well plates by seeding tumor cells and activated CD4^+^ T cells at a 1:10 ratio. After 48 h, CD4^+^ T cells were retrieved for flow cytometry analysis.

### qPCR

Total RNAs were extracted using the RNeasy Mini Kit (Qiagen). First‐strand cDNAs were prepared using 250 ng of RNA and SuperScript II Reverse Transcriptase in the presence of random primers (ThermoFisher Scientific) according to the manufacturer's protocol. Quantitative PCR was performed using an Applied Biosystems QuantStudio 3 System (annealing temperature 60 °C) and PowerUp SYBR Green Master Mix (ThermoFisher Scientific). As described previously,^[^ ^49^ ^]^ all the real‐time values were averaged and compared using the threshold cycle (CT) method, where the amount of target RNA (2^−ΔΔCT^) was normalized to the endogenous expression of *18S* (ΔCT). Primers were purchased from Sigma–Aldrich (KiCqStart SYBR Green Primers) and Biomers. Primers were listed in Table S1 (Supporting Information).

### Western Blotting

Total protein extracts were prepared using RIPA lysis buffer (50 mm Tris, pH 7.5, 150 mm NaCl, 1% Nonidet P‐40, 0.5% sodium deoxycholate, 0.1% SDS, and commercial protease and phosphatase inhibitor cocktail tablets (Roche)). Protein extracts (15‐25 µg) were subjected to electrophoresis on SDS/PAGE. The separated proteins were transferred onto PVDF membranes (Millipore) by electroblotting. Western blots were revealed using the SuperSignal West Dura Extended Duration Substrate (Pierce). Primary and secondary antibodies were listed in Table S2 (Supporting Information). Coomassie blue stainings were performed to visualize equal loading. Loading controls of the blots were shown in Figure S7 (Supporting Information).

### Immunostaining

For immunocytology, cells were grown on glass coverslips, fixed in cold 4% formaldehyde, and permeabilized with 0.05% Tween 20 for 20 min before immunofluorescence experiments. Cells were counterstained with DAPI. All histological experiments were performed as previously described.^[^ ^46^ ^]^ or according to standard protocols on formalin‐fixed paraffin‐embedded tissues. Primary and secondary antibodies were listed in Table S2 (Supporting Information). Brightfield and immunofluorescence images were acquired at ambient temperature using a Plan‐Apochromat 20×/0.8 M27 objective mounted on a Zeiss AxioObserver.Z1/7 microscope equipped with AxioCam 506 color camera and AxioCam 702 mono cameras (Zeiss). For immunofluorescence, sections were counterstained with DAPI. Acquired pictures were subsequently analyzed using ImageJ/Fiji software. All quantifications were performed on at least five random pictures. Background subtraction was performed through setting parameters for color thresholding and circularity. Additionally, for all analyzed pictures, the ImageJ‐generated masks used for quantification were carefully checked by eye to exclude artifacts and false positive areas. Analysis of mouse PDAC pathology was performed by a board‐certified pathologist (T.F.E.B.) in a blinded fashion. For all the representative images, figure panels were cropped from full‐field images and only global, linear brightness/contrast adjustments were applied. No cloning, splicing, or non‐linear transformations were used.

### Tissue Microarray of Human PDAC

Cohort samples consisted of routine diagnostic, formalin‐fixed paraffin‐embedded material from the Institute of Pathology of Ulm University Hospital, originally published in 2015.^[^ ^26^, ^50^ ^]^ The cohort consists of a consecutive series of primary tumor biopsy samples from patients diagnosed between March 2009 and March 2013. Procedures building up this PDAC cohort were carried out with approval from our local ethics committee (ethics #: 255/14). Tumor typing and grading followed WHO criteria^[^ ^51^ ^]^ and staging was performed according to established American Joint Committee on Cancer/TNM criteria (http://www.cancerstaging.org; last accessioned Dec. 19th, 2013). All 105 tumor samples analyzed in this study were treatment‐naïve. Seventeen patients were excluded because of the absence of malignant tissue in formalin‐fixed paraffin‐embedded tumor specimens. DKK3 expression level within the tumor compartment and the fibroblasts was evaluated by a board‐certified pathologist (P.M.). DKK3 expression level was scored from 0 (negative) to 3 (intense) and correlated to patient clinical characteristics.

### Bulk RNA Sequencing

The amount of total RNA was quantified using the Qubit 2.0 Fluorometric Quantitation system (ThermoFisher Scientific) and the RNA integrity number was determined using the 2100 Bioanalyzer instrument (Agilent). RNA‐seq libraries were prepared with the NEBNext Ultra II Directional RNA sample preparation kit (New England Biolabs). Next‐generation sequencing (NGS) library concentrations were quantified with the Qubit 2.0 Fluorometric Quantitation system (Life Technologies) and the size distribution was assessed using the 2100 Bioanalyzer instrument (Agilent). For sequencing, samples were diluted and pooled into multiplex NGS libraries in equimolar amounts. Expression profiling libraries were sequenced on a NovaSeq 6000 instrument (Illumina) following a 50‐base‐pair, paired‐end recipe. NGS reads were mapped to the Genome Reference Consortium GRCm39 assembly via “Spliced Transcripts Alignment to a Reference” (STAR, 2.7.9a).^[^ ^52^ ^]^ utilizing the “basic” GENCODE transcript annotation from version M32 (February 2023) as the reference transcriptome. Since the mm39 assembly flavor of the UCSC Genome Browser was preferred for downstream data processing with Bioconductor packages for entirely technical reasons, GENCODE transcript annotation had to be adjusted to UCSC Genome Browser sequence region names. STAR was run with options recommended by the ENCODE project. NGS read alignments overlapping Ensembl exon features were counted with the Bioconductor (3.16) GenomicAlignments (1.34.0) package via the summarizeOverlaps function in Union mode, ignoring secondary alignments and alignments not passing vendor quality filtering. Since dUTP‐based RNA‐seq protocols lead to the sequencing of the first strand, all alignments needed inverting before strand‐specific counting in feature (i.e., gene, transcript, and exon) orientation. Exon‐level counts were aggregated to gene‐level counts and the Bioconductor DESeq2 (1.38.0) package.^[^ ^53^ ^]^ was used to test for differential expression based on a model using the negative binomial distribution. Biologically meaningful contrasts were extracted from the model, log_2_‐fold values were shrunk with the CRAN ashr (2.2.‐54) package,^[^ ^54^ ^]^ while two‐tailed *P* values obtained from Wald testing were adjusted with the Bioconductor Independent Hypothesis Weighting (IHW, 1.26.0) package.^[^ ^55^ ^]^ The resulting gene tables were annotated and subsequently filtered for significantly differentially up‐ and down‐regulated genes, and subjected to gene set enrichment analysis.

### Gene Term and Gene Set Enrichment Analysis

Gene term enrichment analyses were conducted using the annotation tool DAVID v6.8 (https://davidbioinformatics.nih.gov) with the Gene Ontology, KEGG, and Reactome datasets, and the data sets from the Molecular Signatures Database v6.2 (MSigDB, Broad Institute; http://software.broadinstitute.org/gsea/msigdb). Significant enrichments were defined with *P* ≤ 0.05. Gene set enrichment was performed using GSEA software (v4.3.3). Prior to analysis, a ranked list was calculated with each gene assigned a score based on the sign (“+” or “−”) of log_2_ (FC), multiplied by negative log_10_ (*P* value). GSEA was then performed on the following pathway sets from mSigDB,^[^ ^56^ ^]^ M2. CP – Biocarta, Reactome, Wiki pathways, GO MF for the analysis of the bulk RNA‐seq on acinar cells, and from GO BP, GO MF, and Hallmark for the analysis of the bulk RNA‐seq on tumor cells. Gene sets of the MAPK signaling pathway were obtained from Harmonizome 3.0.^[^ ^57^ ^]^

### Processing of Publicly Available PDAC Single‐Cell RNA Sequencing Data Set

The scRNAseq dataset from Peng and colleagues.^[^ ^27^ ^]^ consisting of 24 PDAC and 11 healthy samples, was obtained from the Genome Sequence Archive (PRJCA001063). Data reprocessing was conducted using Scanpy.^[^ ^58^ ^]^ v1.9.1 in Python. Cells were filtered to retain those with fewer than 10% mitochondrial genes and a maximum of 8000 genes. The raw count data were normalized and log‐transformed. The top 2000 variable features were used to scale the data and perform PCA on the first 50 principal components. Nearest neighbor search and UMAP construction were carried out using the first 20 dimensions. Cell‐type annotations were adopted from the original authors’ information. For downstream analysis, the resulting AnnData object was converted into a Seurat object using SeuratDisk v0.0.0.9021. Analysis of *DKK3* expression and associated visualization was performed using Seurat.^[^ ^59^, ^60^, ^61^, ^62^ ^]^

Single‐cell RNA sequencing data from Carpenter and colleagues^[^ ^40^ ^]^ was obtained from the Shiny application (https://pascadimaglianolab.shinyapps.io/SC_Pancreas_Atlas/).

### Analysis of Publicly Available TCGA Databases

Gene expression information from the TCGA TARGET GTEx study (n = 167 samples from the GTEx pancreas cohort and n = 179 samples from the TCGA‐PAAD cohort) was obtained from UCSC Xena browser (https://xenabrowser.net/). Matching clinical data for the TCGA‐PAAD cohort were obtained from the cBioPortal platform (http://www.cbioportal.org/). Grading data was only available for 177 samples. For pathway enrichment analysis in human PDAC, raw gene expression data from the TCGA‐PAAD cohort were obtained through TCGABiolinks.^[^ ^63^ ^]^ Counts were normalized, and a variance‐stabilizing transformation was applied using DESeq2.^[^ ^53^ ^]^ Enrichment of gene signatures was calculated using gene set variation analysis.^[^ ^64^ ^]^ and correlated with *DKK3* expression.

### Statistical Analysis

Prism software (GraphPad) was used for statistical analysis and graphical representation of the data. Statistical significances were tested using an unpaired Student's t‐test. For survival comparisons, statistical significances were tested using the log‐rank (Mantel‐Cox) test. All tests were considered statistically significant when *P* < 0.05

## Conflict of Interest

L.P. reports nonfinancial support from Ipsen, personal fees from AstraZeneca and Servier outside the submitted work. N.T.G. reports research support from Janssen‐Cilag/Johnson & Johnson, as well as personal fees from AstraZeneca, Daiichi‐Sankyo, J&J, and BMS outside the submitted work. T.S. reports grants and personal fees from Celgene and Sanofi, personal fees from Amgen, AstraZeneca, Bayer, the Falk Foundation, Lilly, Merck‐Serono, Merck, Pierre Fabre, Roche, Servier, and Shire, and grants from Boehringer Ingelheim outside the submitted work. A.K. reports personal fees from the Falk foundation and Amgen outside the submitted work. The other authors declare that they have no competing interests.

## Author Contributions

F.A., J.G., A.K. contributed equally and jointly supervised this work. D.S., F.A., J.G., and A.K. were responsible for the conceptualization of the study. D.S., F.A., J.G., and A.K. defined the methodology. D. Srinivasan, L.P., J.P.M., A.H., S.E.W., N.F., M.K.M., and F.A. conducted experiments. D.S., E.R., E.Z., T.F.E.B., P.M., C.A., and F.A. conducted formal analysis of the data. D.S., E.R., E.Z., and C.A. performed data visualization. N.T.G., P.M., D.G., T.S., and S.L. provided resources. J.‐M.L. provided the pancreatic stellate cell line. D.S., N.A., J.G., and A.K. wrote the original draft of the manuscript. T.S., N.A., and A.K. were responsible for project administration. J.G. and A.K. were responsible for the acquisition of funding.

## Supporting information



Supporting Information

## Data Availability

The raw and processed RNA‐seq data generated for this project are available on GEO with accession numbers GSE283238 and GSE307602.
