# Supplementary material for: DKK3 Initially Preserves Acinar Integrity Through MEK‐Fos Signaling, but Later Switches to an Oncogenic Role in Pancreatic Cancer
Source: Adv Sci (Weinh). 2025 Oct 28;13(3):e17606. doi: 10.1002/advs.202417606 (PMC12806428; doi:10.1002/advs.202417606)
Supplement: Supplementary file 1 — Supporting Information [file ADVS-13-e17606-s001.docx]

**DKK3 initially preserves acinar integrity through MEK-Fos signaling, but later switches to an oncogenic role in pancreatic cancer**

**Short informative title:**

DKK3 preserves acinar integrity at early stages but later promotes aggressiveness in advanced stages in pancreatic cancer

**Short running title:**

DKK3 as a double-edged sword in pancreatic cancer

Dharini Srinivasan^1^, Elodie Roger^1^, Lukas Perkhofer^1,2^, Eleni Zimmer^1^, Thomas F.E. Barth^3^, Julia P. Mosler^1^, Anna Härle^1^, Stephanie E. Weissinger^3^, Nadine T. Gaisa^3^, Peter Möller^3^, Nico Fischer^4,5^, Chantal Allgöwer^1^, J.-Matthias Löhr^6^, Dirk Grimm^4,5,7^, Thomas Seufferlein^8^, Stefan Liebau^9^, Ninel Azoitei^1^, Michael K. Melzer^1,10^, Frank Arnold^11,#^, Johann Gout^1,#^, Alexander Kleger^1,2,12,#^

^1^Institute for Molecular Oncology and Stem Cell Biology, Ulm University Hospital, Ulm, Germany

^2^Division of Interdisciplinary Pancreatology, Department of Internal Medicine I, Ulm University Hospital, Ulm, Germany

^3^Institute of Pathology, Ulm University Hospital, Ulm, Germany

^4^Department of Infectious Diseases/Virology, Section Viral Vector Technologies, Medical Faculty, Heidelberg University, Heidelberg, Germany.

^5^BioQuant, BQ0030, Heidelberg University, Heidelberg, Germany.

^6^Department of Clinical Science, Intervention and Technology (CLINTEC), Pancreas Cancer Research Lab, Karolinska Institute, Stockholm, Sweden

^7^German Center for Infection Research (DZIF) and German Center for Cardiovascular Research (DZHK), partner site Heidelberg, Heidelberg, Germany.

^8^Department of Internal Medicine I, Ulm University Hospital, Ulm, Germany

^9^Institute of Neuroanatomy & Developmental Biology INDB, Tübingen, Germany

^10^Department of Urology, Ulm University Hospital, Ulm, Germany

^11^Cancer Biology & Genetics Program, Memorial Sloan Kettering Cancer Center, New York, NY, USA

^12^Core Facility Organoids, Ulm University, Ulm, Germany

**Corresponding author**

Prof. Dr. Alexander Kleger

Institute of Molecular Oncology and Stem Cell Biology (IMOS), Ulm University Hospital

Albert-Einstein-Allee 23, 89081 Ulm, Germany

Phone: +49-731-500-44728 Fax: +49-731-500-44612

Email: [alexander.kleger@uni-ulm.de](mailto:alexander.kleger@uni-ulm.de)

**List of Supplementary Materials**

**Supplementary Experimental section.**

**Supplementary Table 1:** List of primers used for qPCR analysis.

**Supplementary Table 2:** List of antibodies used for western blot analysis and immunostainings.

**Supplementary Figure S1:** Loss of DKK3 leads to aggressiveness and rapid tumorigenesis.

**Supplementary Figure S2:** DKK3 safeguards acinar integrity and restrains dysplasia during PDAC onset.

**Supplementary Figure S3:** Fos orchestrates tumor-promoting effects of DKK3 loss.

**Supplementary Figure S4:** DKK3-null tumors maintain their aggressive phenotype by reshaping the tumor microenvironment.

**Supplementary Figure S5:** IL6-JAK-STAT3 signaling mediates the oncogenic activity of DKK3.

**Supplementary Figure S6: DKK3-expressing fibroblasts acts in an oncogenic manner at end-stage PDAC.**

**Supplementary Figure S7: Western blot loading controls.**

**Supplementary experimental section**

**Wound healing assay**

Three hundred thousand tumor cells were seeded in 48-well plate in DMEM containing 10% FBS and P/S. After 24 hours, the tumor cell layer was scratched using sterile tips and the culture medium was replaced with serum-free DMEM containing P/S for 48 h. Brightfield images were captured after 0 and 8 hours, using a Zeiss Axio Imager Z1 fluorescence microscope equipped with an AxioCam MRm camera and AxioVision LE imaging software (Zeiss). Acquired pictures were subsequently analyzed using ImageJ software.

For the Incucyte (Sartorius) Scratch Wound assay, 200,000 tumor cells were seeded in Incucyte Imagelock plates. Tumor cells were pre-treated with 20 μg/mL rDKK3 (Sino Biological) or 20 µM T5224 (Selleckchem) for 72 hours and then seeded in the Incucyte Imagelock plate. Wound was generated using Incucyte Woundmaker Tool. Cells were further treated with 20 μg/mL rDKK3 or 20 µM T5224. Images were taken at regular intervals by the Incucyte live-cell analysis system for up to 48 hours. The analysis was done using the integrated analysis algorithm under Incucyte Scratch Wound Analysis Software Module.

**Migration assay**

For transwell migration assay, 200,000 cells were seeded in serum-free medium in the upper chamber of transwells with 8 μm pore size membrane (24-well format, Falcon) and complete medium in the lower chamber. Tumor cells were pre-treated with rDKK3 (20 μg/mL) for 72 hours, then seeded in 24-well plates and treated further before the start of the experiment. For treatment with the STAT3 inhibitor stattic (Selleckchem), rDKK3-pre-treated tumor cells were seeded in 24-well plates and further treated with rDKK3 and stattic for 48 hours. After 48 hours, cells were fixed with cold 4% formaldehyde and stained with 5% Giemsa. Cells migrating to the membrane lower side were counted using ImageJ software (National Institutes of Health).

**P-ERK ELISA**

## Protein lysates were collected from WT and DD acinar cells after three days of *ex vivo* ADM assay. ELISA was carried out according to manufacturer’s instructions (mouse PERK ELISA kit, Novus Biologicals).

**Cell viability assay**

**Cells were seeded in 96-well plates (2,000 per well). Cells were treated for three days, 24 hours after seeding. Cell viability was analyzed with an MTT assay (Sigma-Aldrich) according to the manufacturer’s protocol. Absorbance was measured at 590 nm wavelength using a spectrophotometer (Tecan Infinite M200 Pro). Viability percentages were normalized to vehicle-treated cell viability. Half maximal inhibitory concentrations were determined by Prism software (GraphPad).**

**Flow cytometry**

Staining of CD4^+^ T cells was performed in FACS buffer (PBS, 0.3% (w/v) BSA, and 0.1% (w/v) NaN_3_). Non-specific binding of antibodies to Fc-receptors was blocked by pre-incubating cells with mAb 2.4G2 (BD Biosciences) directed against the FcγRIII/II CD16/CD32 (0.5 µg mAb/10^6^ cells/100 µL). Surface staining was performed using CD4 (RM4-5) APC-conjugated and CD25 (PC61.5) PE-Cy7-conjugated monoclonal antibodies (ThermoFisher Scientific). Intracellular FoxP3 staining was performed using eBioscience FoxP3/Transcription Factor Staining Buffer Set following manufacturer’s protocol. Flow cytometry was performed with LSRII (BD Biosciences) or AttuneTM NxT (ThermoFisher Scientific) flow cytometers. Results were analyzed with FlowJo software (BD Biosciences).

**Syngeneic orthotopic transplantation**

For orthotopic transplantations, tumor cells were implanted by an injection of 25 × 10^3^ cells in 100 µL of 1:1 serum-free DMEM:Matrigel GFR into the pancreas of eight-week-old *Dkk3*^+/+^ and *Dkk3*^-/-^ mice (n = 3 for *Dkk3*^+/+^ mice; n = 2 for *Dkk3*^-/-^ mice). Tumor take was 100% for each cell line. Tumors were resected and fixed in cold 4% formaldehyde for 24 hours and embedded in paraffin for histological analysis.

**Supplementary Tables**


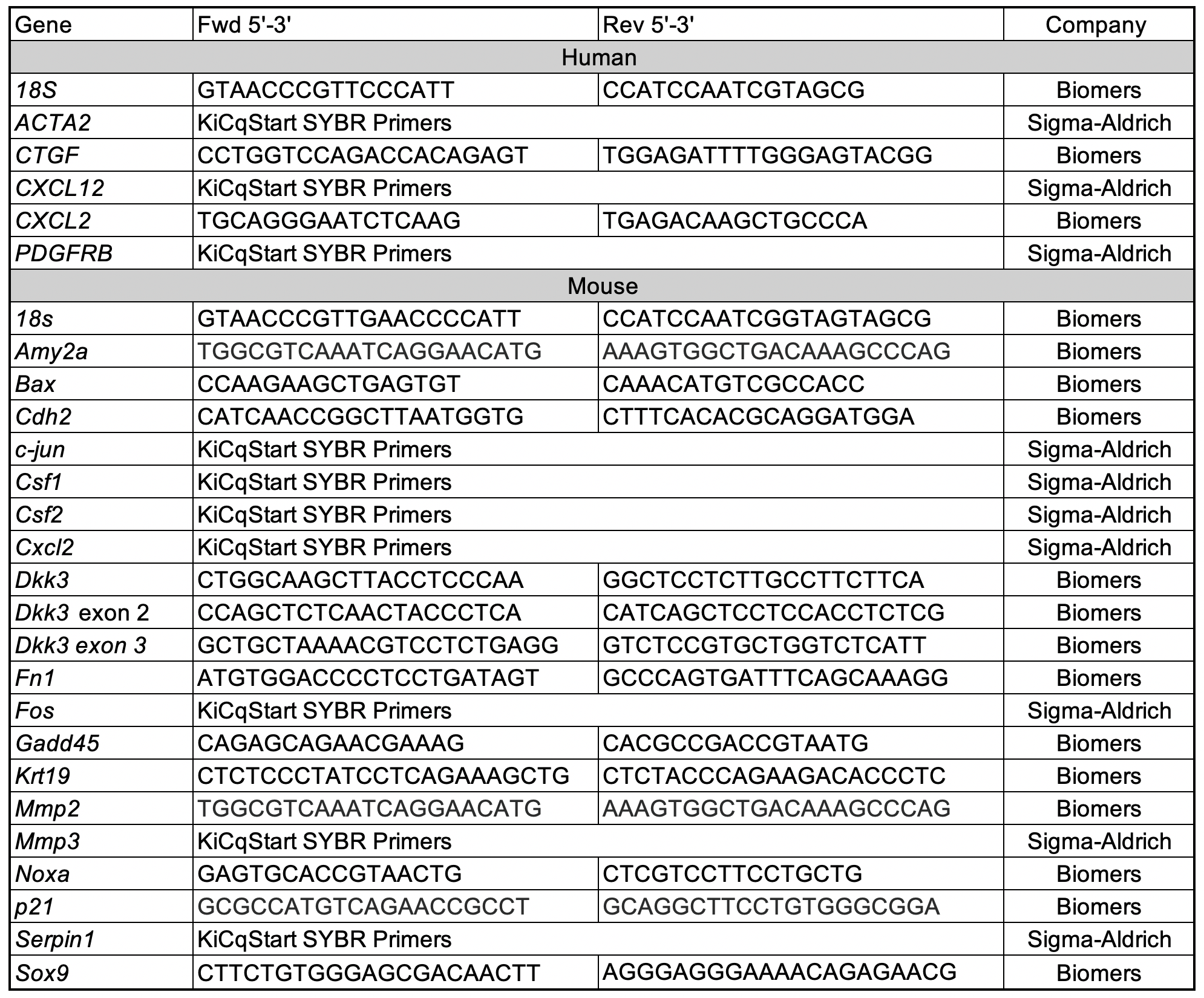
**Supplementary Table 1. List of primers used for qPCR analysis.**

**Supplementary Table 2. List of antibodies used for western blot analysis and immunostainings.**


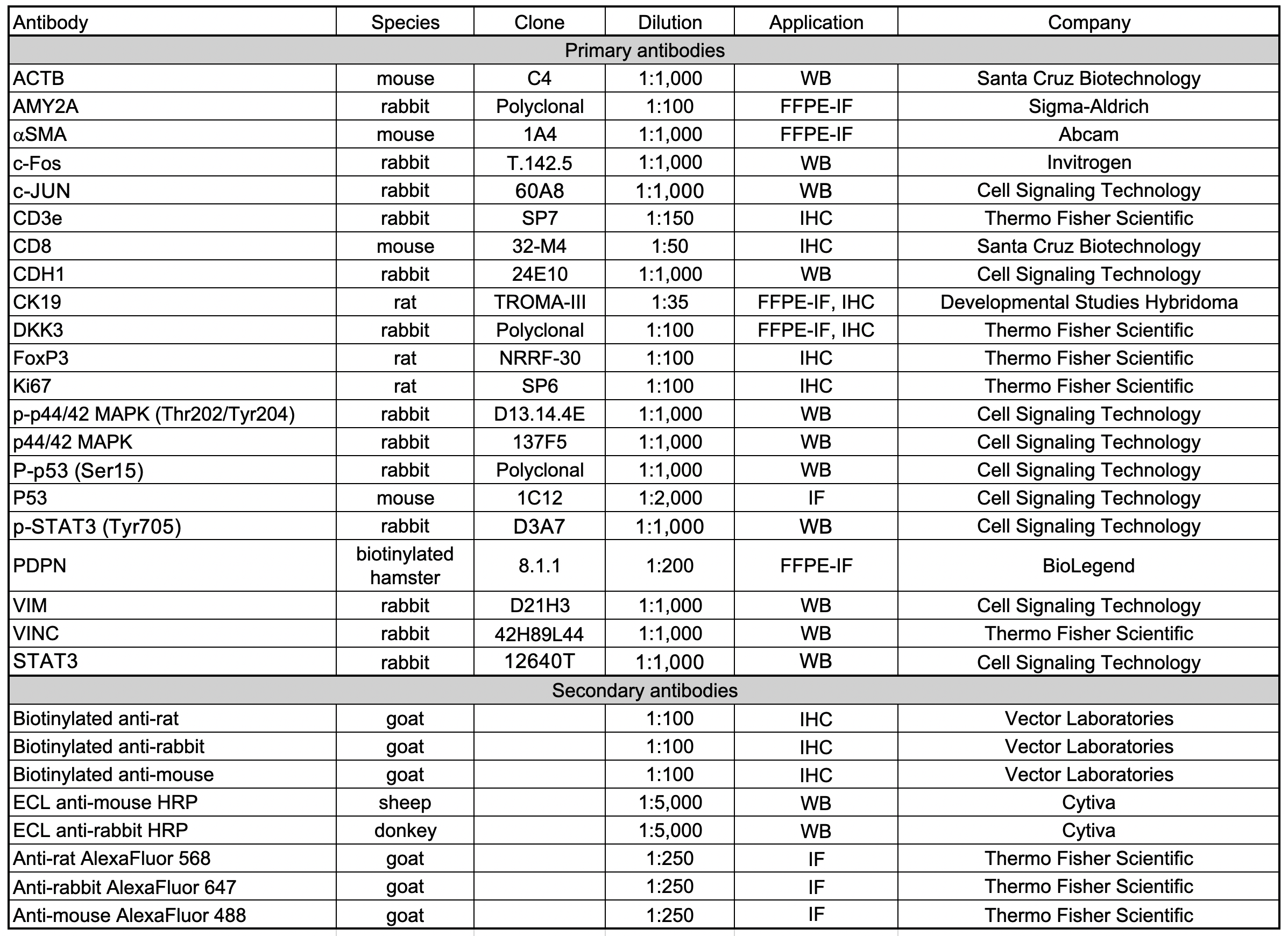
FFPE, formalin-fixed paraffin-embedded; IF, immunofluorescence; IHC, immunohistochemistry; WB, western blotting.

**Supplementary Figure and Legends**

**
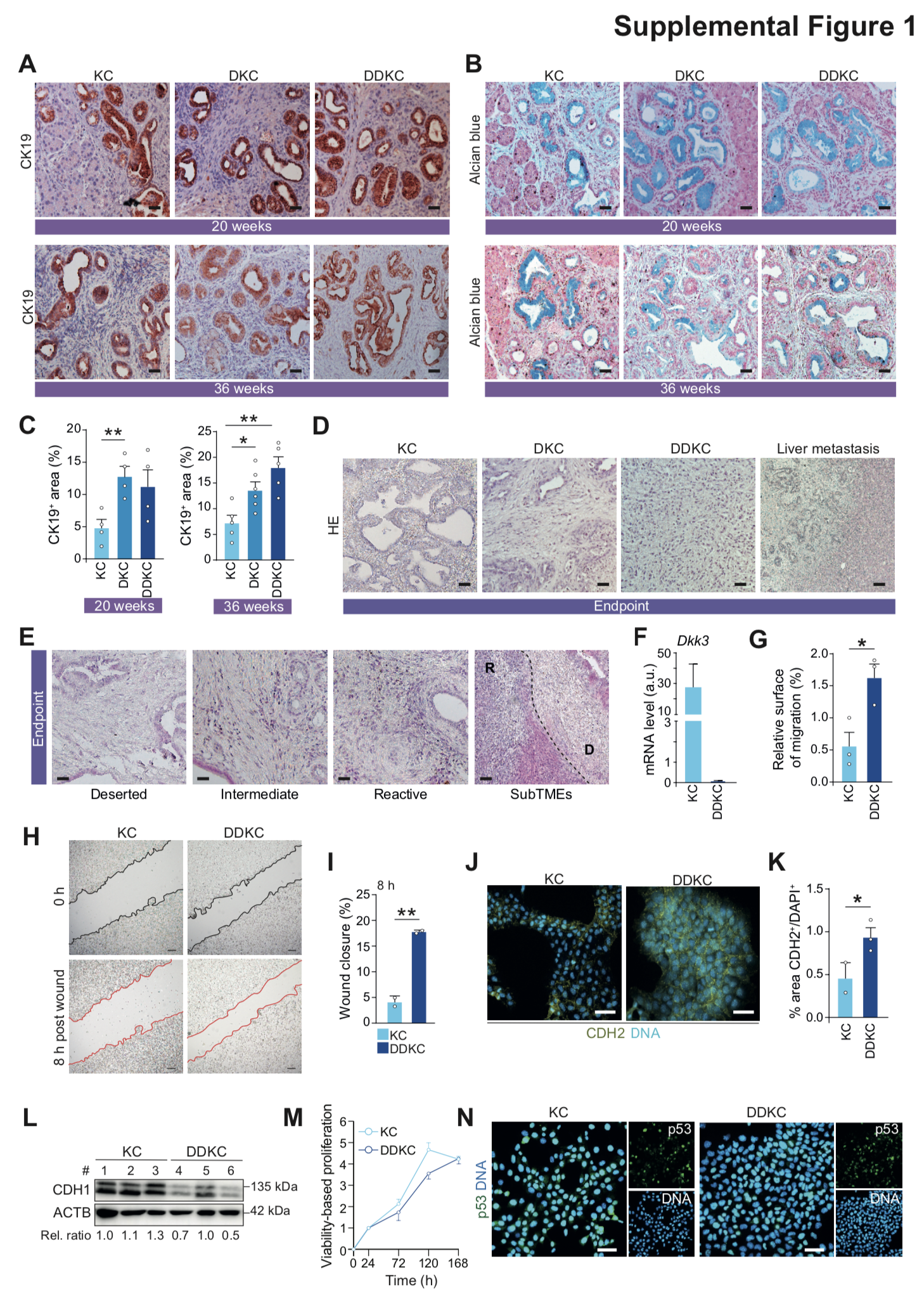
**

**Supplementary Figure S1. Loss of DKK3 leads to aggressiveness and rapid tumorigenesis.**

(**A** and **B**) Immunohistochemistry staining for CK19 (A) and alcian blue staining (B) on pancreatic sections from intermediate time point. Scale bar, 50 µm. (**C**) Quantification of CK19^+^ area in intermediate time point pancreata. (**D**) Hematoxylin-eosin (HE) staining on histological sections of pancreata at endpoint. Representative image of a liver metastasis at endpoint stained with HE (right panel). Scale bar, 50 µm. (**E**) Representative images of various subTMEs quantified in Fig. 1M, stained by HE. Scale bar, 50 µm. Dotted lines represent specific areas of various subTMEs. (**F**) qRT-PCR analysis of *Dkk3* expression in tumor cells. (**G**) Relative surface of migration of tumor cells after 48 hours of Boyden chamber assay. (**H**) Representative images of wound healing assay on KC and DDKC tumor cells, 0 and 8 hours post wound generation. Scale bar, 50 µm. (**I**) Wound closure of KC and DDKC tumor cells. (**J**) Immunofluorescence for CDH2 on tumor cells. Scale bar, 50 µm. (**K**) Quantification of CDH2^+^ area (relative to the total nucleus count). (**L**) Western blot analysis and quantification of CDH1 levels in tumor cells. (**M**) Viability-based proliferation assay of KC and DDKC tumor cells. (**N**) Immunofluorescence staining for P53 (green) in tumor cells. Scale bar, 50 µm. Data are means ± SEM. Each dot represents a mouse (C) or a cell line (G, I, K). Significance was calculated by unpaired Student’s t-test. **P* < 0.05; ***P* < 0.01. D, deserted; R, reactive.


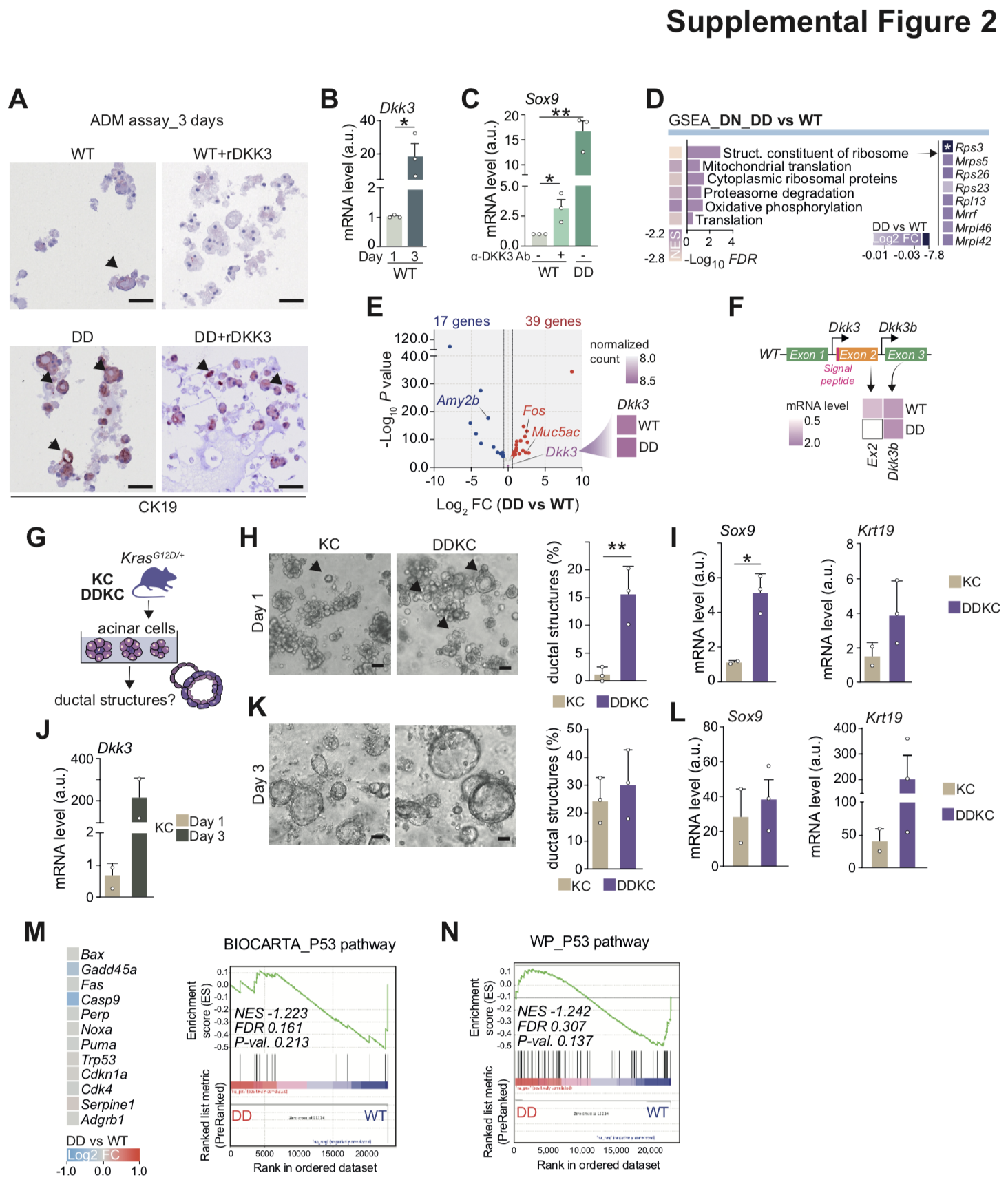


**Supplementary Figure S2. DKK3 safeguards acinar integrity and restrains dysplasia during PDAC onset.**

(**A**) Immunohistochemistry staining for CK19 on WT and DD acini, treated or not with rDKK3 after 3 days of ADM assay. Scale bar, 50 µm. (**B**) qRT-PCR analysis of *Dkk3* expression in WT acinar cultures at ADM assay day 1 and day 3. (**C**) qRT-PCR analysis of ductal marker gene *Sox9* in WT (treated or not with neutralizing anti-DKK3 antibody) and DD acinar cultures after three days of ADM assay. (**D**) GSEA of the transcriptomics data showing downregulated pathways with *FDR* ≤ 0.05 in DD versus WT acinar cultures. Heatmap represents log_2_ FC of top genes within the indicated gene sets. (**E**) Volcano plot displaying differential expressed genes between DD and WT acinar cells (left panel). Red dots represent the upregulated expressed transcripts and blue dots represent the downregulated expressed transcripts. Normalized count of *Dkk3* expression in WT and DD acini cultures. (**F**) Schematic representation of the *Dkk3* wild-type allele, and exon-specific qRT-PCR analysis of *Dkk3 exon 2* and *Dkk3b* in WT and DD acini cultures. (**G**) Schematic representation of experimental setup (H-L). (**H**) Brightfield images of KC and DDKC acinar cultures at day 1 of ADM assay and quantification of ductal structures in acinar cultures after 24 hours of ADM assay. Arrows show ductal structures. Scale bar, 50 µm. (**I**) qRT-PCR analysis of *Sox9* and *Krt19* expression in KC and DDKC acinar cultures at day 1 of ADM assay. (**J**) *Dkk3* expression in KC acinar cultures at ADM assay day 1 and day 3. (**K**) Brightfield images of KC and DDKC acinar cultures at day 3 of ADM assay. Arrows show ductal structures. Scale bar, 50 µm. (**L**) qRT-PCR analysis of *Sox9* and *Krt19* expression in KC and DDKC acinar cultures at day 3 of ADM assay. (**M** and **N**) GSEA of the P53 pathway gene set in BIOCARTA (M) and WikiPathway (N). Heatmap represents log_2_ FC of top genes within the indicated gene sets. Data are means ± SEM. Each dot represents a mouse (B, C, H-L). ADM, acinar-to-ductal metaplasia; DN, downregulated; Fc, fold change; FDR, false discovery rate; NES, normalized enrichment score.


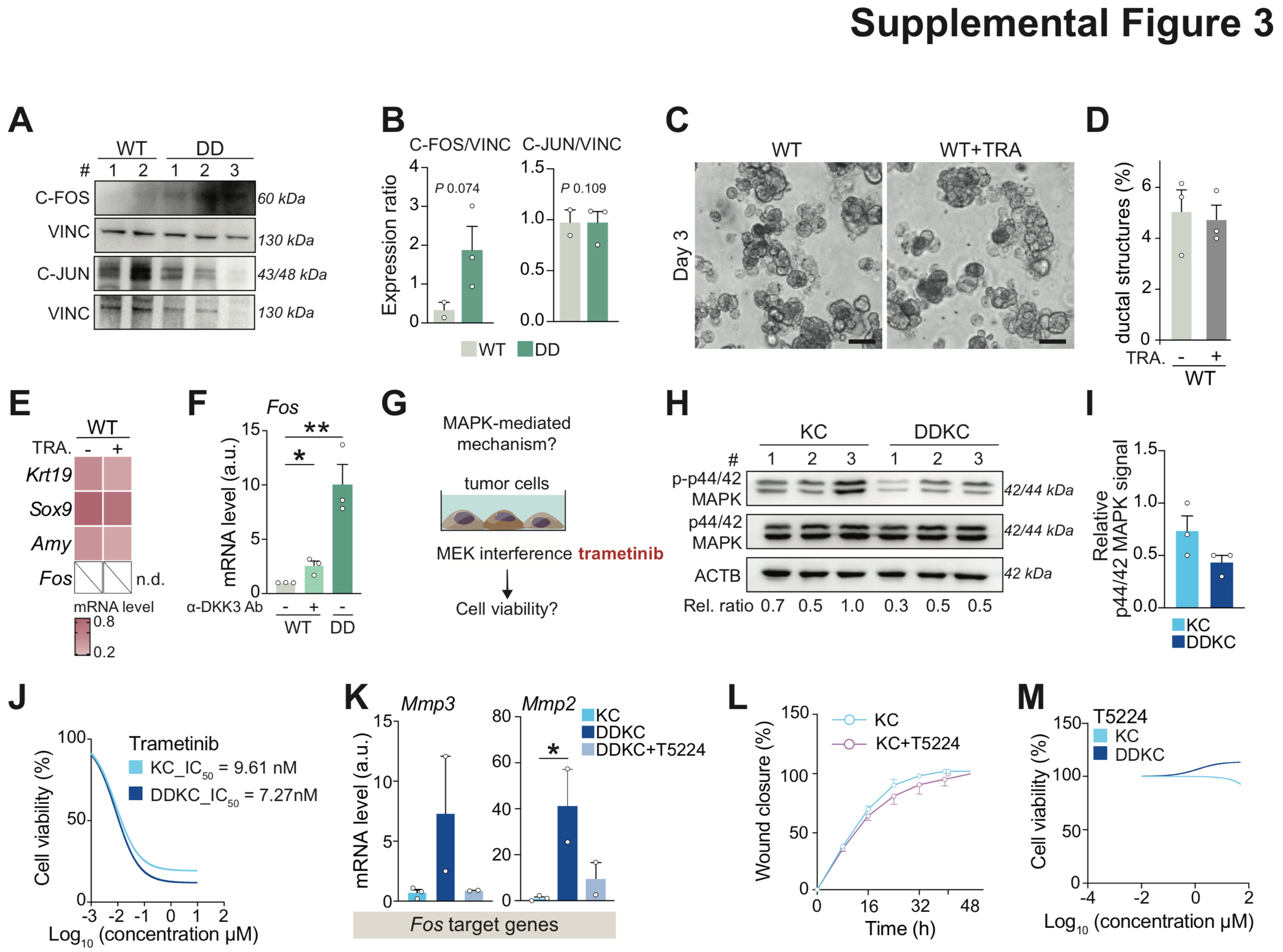


**Supplementary Figure S3. Fos orchestrates tumor-promoting effects of DKK3 loss.**

(**A** and **B**) Western blot analysis (A) and quantification (B) of Fos and C-Jun in WT and DD acinar cultures after three days of ADM assay. (**C** and **D**) Brightfield images (C) and quantification of ductal structures (D) in WT acinar cultures treated or not with the MEK1/2 inhibitor trametinib for three days. Scale bar, 50 µm. (**E**) qRT-PCR analysis of acinar (*Amy*) and ductal (*Krt19* and *Sox9*) marker genes, and of *Fos* expression in WT acinar cultures treated or not with trametinib for three days. (**F**) qRT-PCR analysis of *Fos* expression in WT treated or not with neutralizing anti-DKK3 antibody and DD acinar cultures after four days. (**G**) Experimental design shown in (H to J). (**H** and **I**) Western blot analysis (H) and quantification (I) of p44/42 MAPK phosphorylation (p-T202/Y204, p-T185/Y187) levels in KC and DDKC tumor cells. (**J**) Viability assay analysis of trametinib treatment in KC and DDKC tumor cells. (**K**) qRT-PCR analysis of Fos target gene expression in KC and DDKC tumor cells treated or not with T5224 for 48 hours. (**L**) Wound closure of KC tumor cells treated or not with T5224. (**M**) Cell viability of KC and DDKC tumor cells treated with T5224. Data are means ± SEM. Each dot represents a mouse (B, D, F) or a cell line (I, K). Significance was calculated by unpaired Student’s t-test. **P* < 0.05. ADM, acinar-to-ductal metaplasia; IC_50_, half maximal inhibitory concentration; n.d., not detected; TRA, trametinib.

**
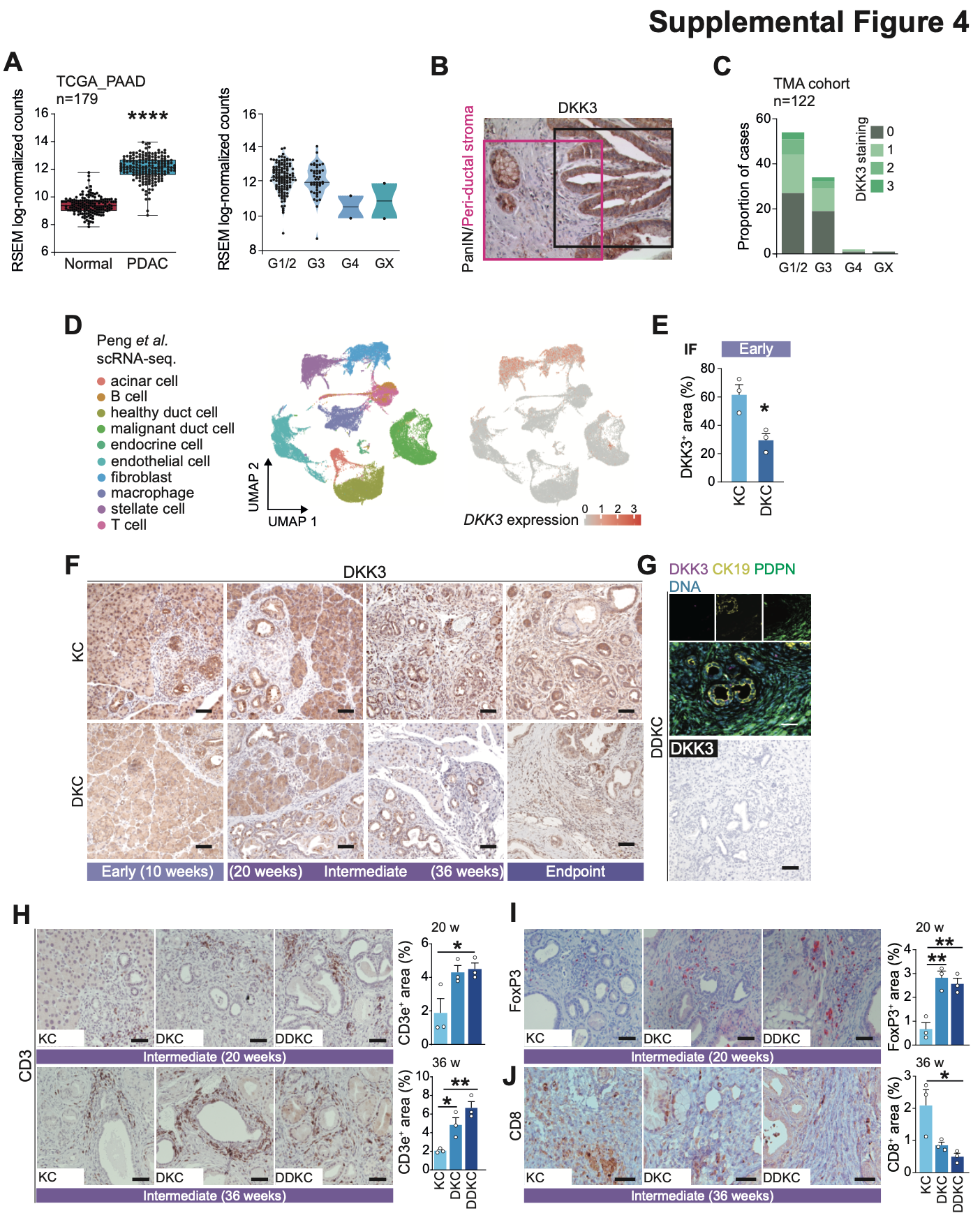
**

**Supplementary Figure S4. DKK3-null tumors maintain their aggressive phenotype by reshaping the tumor microenvironment.**

(**A**) Dot plot representing *DKK3* expression in normal versus cancer tissue in PDAC patients of the TCGA dataset (top panel) and violin plot representing *DKK3* expression in PDAC patients (of the TCGA dataset) based on tumor grading (bottom panel). (**B**) Full-field image of DKK3 immunostaining with black and pink boxes respectively indicating the cropped areas used for the PanIN “3” and “Stroma” panels shown in Figure 4A. Both fields were derived from adjacent regions of the same histological section. **(C)** Proportion of cases under different PDAC grades in human TMA characterized in Figure 4A. (**D**) UMAP obtained from the reanalysis of publicly available single-cell dataset (left panel) **and** *DKK3* expression in the dataset as shown in left panel. (**E**) Quantification of DKK3^+^ area in pancreata at early timepoint. (**F**) **Immuno**histochemistry staining for DKK3 on KC and DKC pancreatic sections from early 10 weeks to endpoint. **(G)** immunofluorescence for DKK3 (purple), CK19 (yellow), and PDPN (green) and immunohistochemistry of DKK3 in DDKC tumors. Scale bar, 50 µm. (**H**) **Immuno**histochemistry staining for CD3 on pancreatic sections from intermediate 20 and 36 weeks and quantification of CD3^+^ area **in intermediate time point pancreata.** Scale bar, 50 µm. **(I) Immuno**histochemistry staining for FoxP3 on pancreatic sections from intermediate 20 weeks and quantification of FoxP3^+^ area. Scale bar, 50 µm. (**J**) **Immuno**histochemistry staining for CD8 on pancreatic sections from intermediate 36 weeks and quantification of CD8^+^ area. Scale bar, 50 µm. Data are means ± SEM. Each dot represents a mouse (D, G, H, I). Significance was calculated by unpaired Student’s t-test. **P* < 0.05; ***P* < 0.01; *****P* < 0.0001. PAAD, pancreatic adenocarcinoma, RSEM, RNA-seq by expectation-maximization; TCGA, The Cancer Genome Atlas; TMA, tissue microarray; UMAP, uniform manifold approximation and projection.


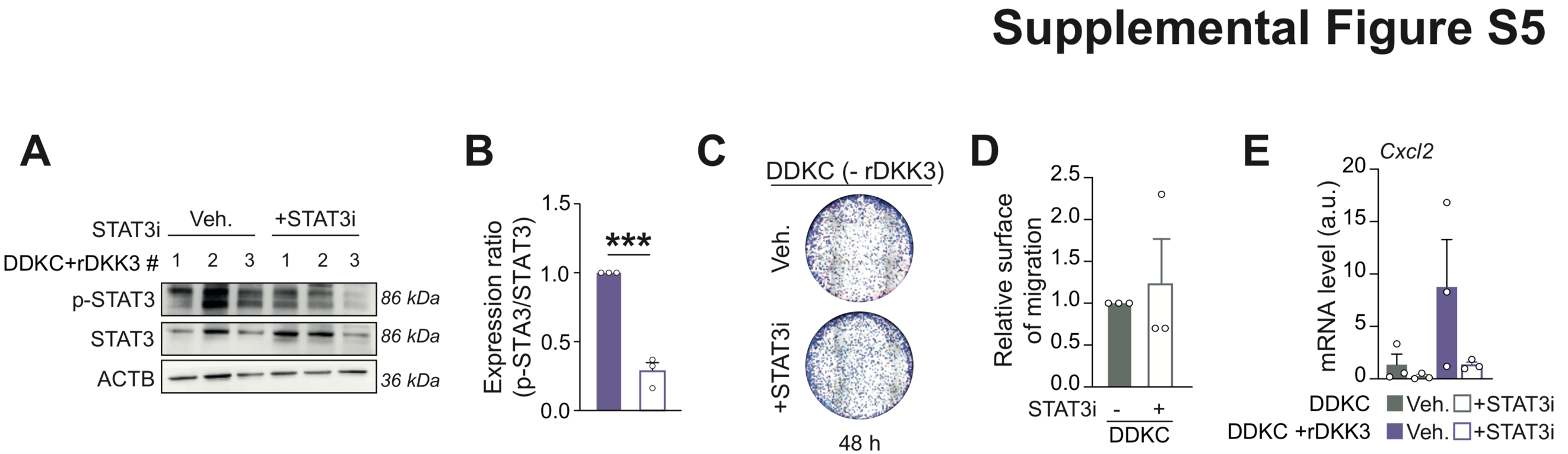


**Supplementary Figure S5. IL6-JAK-STAT3 signaling mediates the oncogenic activity of DKK3.**

(**A** and **B**) Western blot analysis (A) and quantification of p-Y705 STAT3 (p-STAT3) (B) in rDKK3-treated DDKC tumor cells, treated or not with the STAT3 inhibitor (STAT3i) stattic for 48 hours. **(C)** Boyden chamber assay with DDKC tumor cells, treated or not with STAT3i for 48 hours. (**D**) Relative surface of migration of DDKC tumor cells, treated or not with STAT3i for 48 hours. (**E**) qRT-PCR analysis of the IL6-JAK-STAT3 target gene *Cxcl2* in DDKC and rDKK3-treated DDKC tumor cells, treated or not with STAT3i for 48 hours. Data are means ± SEM. Each dot represents a cell line (B, D, E). Significance was calculated by unpaired Student’s t-test. **P* < 0.05; ***P* < 0.01; *****P* < 0.0001.


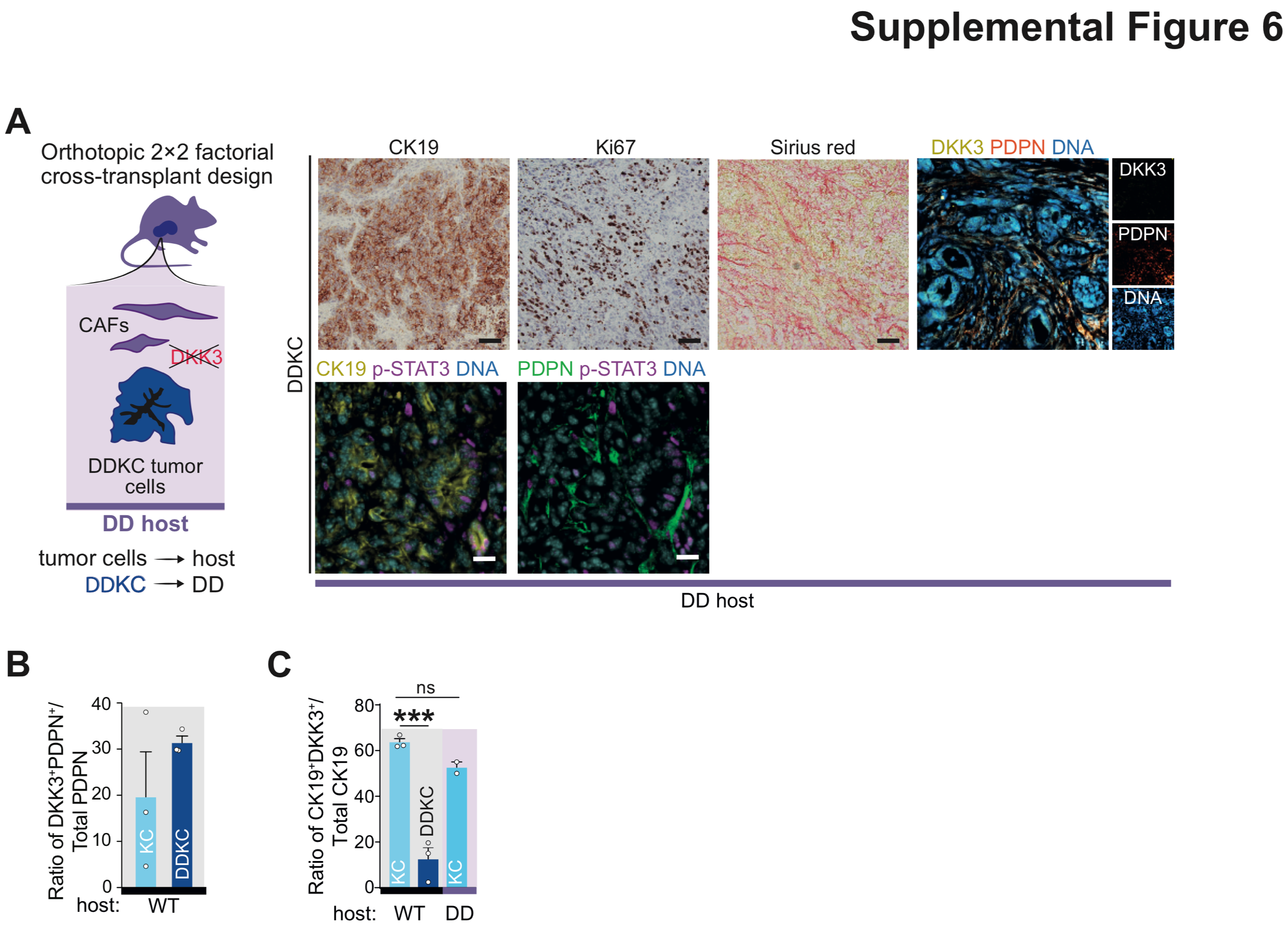


**Supplementary Figure S6. DKK3-expressing fibroblasts acts in an oncogenic manner at end-stage PDAC.**

(**A**) Schematic representation of the experimental design (left) and immunohistochemistry staining for CK19 and KI67, picrosirius red staining, and immunofluorescence for DKK3 (purple), CK19 (yellow), and PDPN (green) and pSTAT3 (purple), CK19 (yellow) and PDPN (green) on sections of resected tumors arising from DDKC tumor cells transplanted in DD host (right). Scale bar, 50 µm. (**B**) Quantification of DKK3^+^PDPN^+^ cells (relative to total PDPN cells) in resected tumors arising from KC and DDKC tumor cells transplanted in WT host. (**C**) Quantification of DKK3^+^CK19^+^ cells (relative to total CK19^+^ cells) in KC and DDKC tumor cells transplanted in WT host and KC tumor cells transplanted in DD host (orthotopic assay shown in Figure 6). Data are means ± SEM. Each dot represents a mouse (A, C). Significance was calculated by unpaired Student’s t-test. ****P* < 0.001.


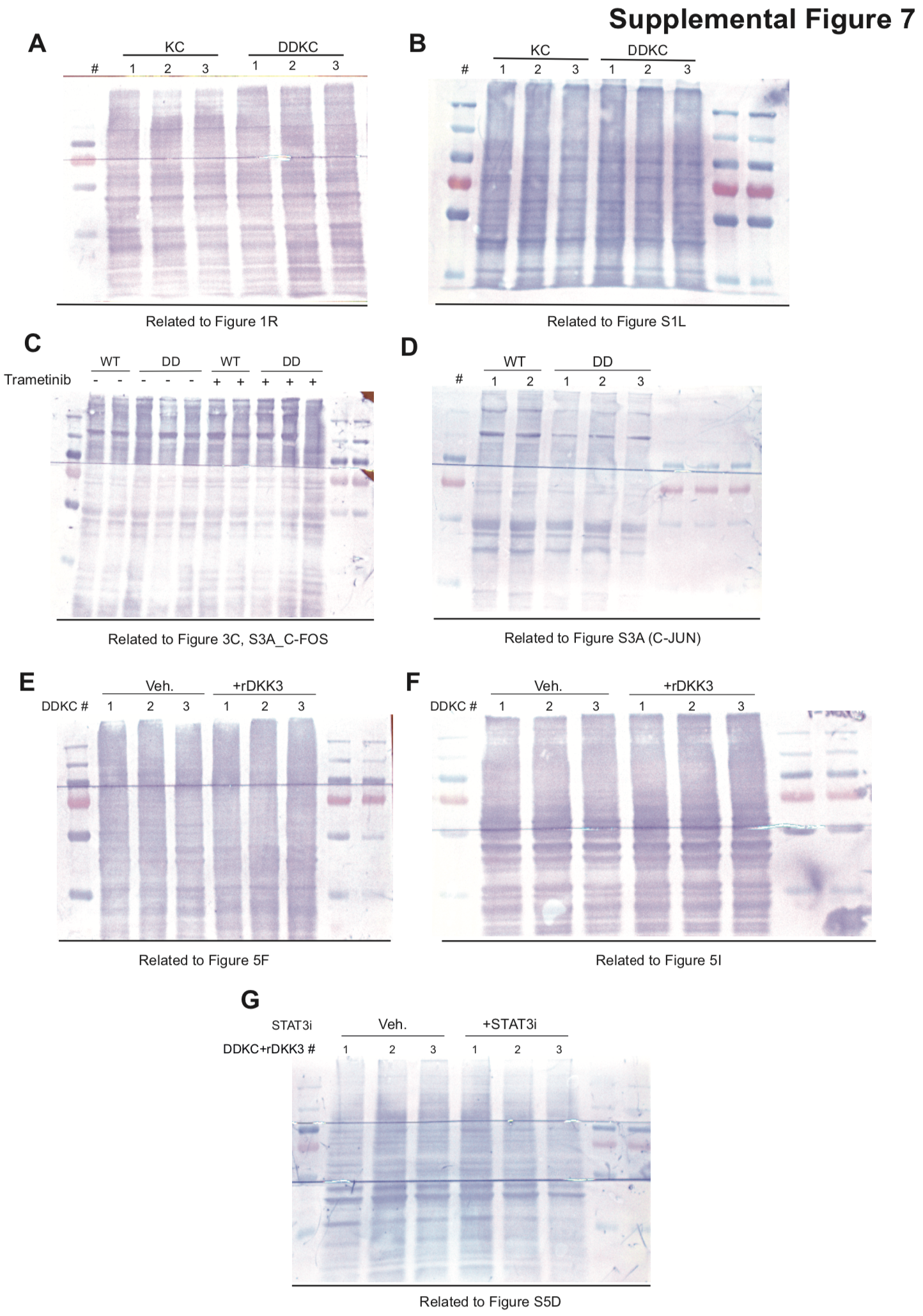


**Supplementary Figure S7. Western blot loading controls.**

(**A**–**G**) Western blot stained with Coomassie blue related to Figure 1R (A), S1L (B), 3C/S3A (C), S3A (D), 5F (E), 5I (F), and S5D (G).
